# Enhancing dance learning by optimising instructional perspective: A preregistered study on the effect of dancers’ viewing perspective, cognitive load and expertise

**DOI:** 10.1371/journal.pone.0355860

**Published:** 2026-09-16

**Authors:** Elisabetta Versace, Manuela Angioi, Kristin Hadfield, Vincent Gallo, Lars Juhl Jensen, Juncal Roman, Karen Sheriff, Emma Redding, Frances Clarke, Julian Deering, Dylan Morrissey, Stephen Buckingham

**Affiliations:** 1 School of Biological and Behavioural Sciences, Centre for Brain and Behaviour, Queen Mary University of London, London, United Kingdom; 2 Sports and Exercise Medicine, William Harvey Research Institute, Queen Mary University of London, London, United Kingdom; 3 Trinity Centre for Global Health, School of Psychology, Trinity College Dublin, Dublin, Ireland; 4 Faculty of Health and Medical Sciences, Novo Nordisk Foundation Center for Protein Research, University of Copenhagen, Copenhagen, Denmark; 5 The English National Ballet School, London, United Kingdom; 6 The Royal Ballet School, London, United Kingdom; 7 Trinity Laban Conservatoire for Music and Dance, London, United Kingdom; 8 Faculty of Fine Arts and Music, Victorian College of Arts, University of Melbourne, Melbourne, Australia; 9 Department of Drama, School of the Arts, Queen Mary University of London, London, United Kingdom; 10 Physiotherapy Department, Mile End Hospital, Barts Health NHS Trust, London United Kingdom; Università degli Studi di Milano: Universita degli Studi di Milano, ITALY

## Abstract

While movement sequences in choreography are often taught through instructor demonstrations, the influence of visual perspective on learning outcomes has received limited attention. Drawing on cognitive load theory, we investigated how the requests imposed by different instructional perspectives affect learning and performance. We compared the effects of Back view (first-person), Front mirror view (third-person mirrored), and Front opposite view (third-person) between dancers. We hypothesized lower cognitive load and better performance in the Back view, particularly for less experienced dancers. After preregistering the study, 32 dancers with varying levels of experience learned a moderately complex choreography from pre-recorded videos, in the three perspectives. Five dance experts assessed movement accuracy, spatial skills, and rhythm/dynamics using a validated scoring system and a new open-source digital tool. The Back view consistently yielded superior outcomes, especially among less trained dancers. No significant differences emerged between the two third-person views, possibly due to sample size or dancer background. These findings suggest that first-person perspectives may support motor learning, with performance patterns consistent with reduced cognitive load. Practically, camera perspective should be considered in online instruction, while first-person views may benefit early in-person training. Our results highlight the role of visual perspective in learning choreography and complex motor routines, offering both theoretical and applied contributions, including validation of a novel digital assessment tool.

## Introduction

Millions of people worldwide learn some form of dance, either recreationally or professionally, from a teacher present in the same room or from screens [1–3]. For all these different approaches it is still not clear what the most effective ways are to teach a piece of choreography, or any sophisticated motor sequence. This study investigates how the different visual perspectives in which a dancer sees the instructor do influence choreography learning and performance. While we focus on dance, similar issues apply to other sports, aesthetic disciplines in particular [4].

In sports settings, training load management has been implemented to optimise performance and reduce the risk of injury [5,6]. While the role of physiological and mechanical load has been emphasized [5,7–9], the impact of cognitive load on learning motor sequences is only starting to be understood. *Cognitive load* refers to the amount memory and other cognitive resources involved in task execution and learning [10].

A high cognitive load can negatively impact learning and performance, as observed in the laboratory [11–13] and in different sports settings. For instance, rowers engaged in solving arithmetic problems reduce movement complexity [14,15], team ball athletes show lower agility when engaged in cognitively demanding tests [16], and dancers reduce postural control when engaged in concurrent mental tasks [17]. The impact of cognitive load on the acquisition of complex motor sequences, such as dance choreographies, is less understood, although we know that expert dancers can chunk movement sequences with lower effort [18] and potentially engage with lower attentional effort than novices [19].

In dance, perceptual, cognitive, motor, emotional and social demands are tightly integrated [20–22], as learners must simultaneously process multimodal information (visual, auditory and proprioceptive) movement sequences, spatial orientation and timing, often under time constraints, artistic demands and social pressure. Thus, understanding conditions that enhance dance learning is relevant not only for the wider field of dance but also to understand the mechanisms underlying complex motor learning more broadly [23]. One of the important factors in play is cognitive load.

Cognitive load builds from different components, including intrinsic and extraneous load. First, the *intrinsic load* refers to the inherent difficulty of a task, which is influenced by the expertise of the learner [24,25]. For instance, choreography that is cognitively demanding for a novice may impose little cognitive load on an expert. We used the current amount of training as a proxy for expertise, as previous studies showed that dancers with more experience are better in chunking and recalling dance sequences [26], and that current practice is strongly associated with dance performance [27]. According to the well-established cognitive load theory of the expertise reversal effect, task demands differentially affect learners based on their existing schemas [24].

**Fig 1:**
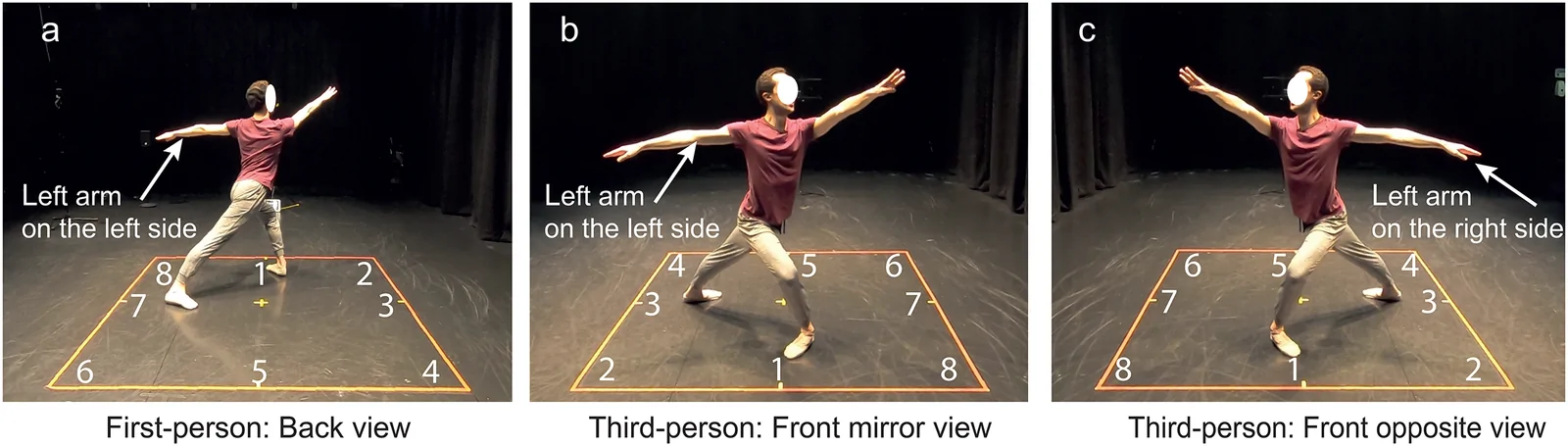
The three different perspectives of the teacher. (a) First person: Back view, where participant could simply follow the choreographer. (b) Third person: Front mirror view: where participants could move the limb on the same right/left side of the choreographer. (c) Third person: opposite view: where participants could move the limb on the same right/left side of the choreographer.

**Fig 2:**
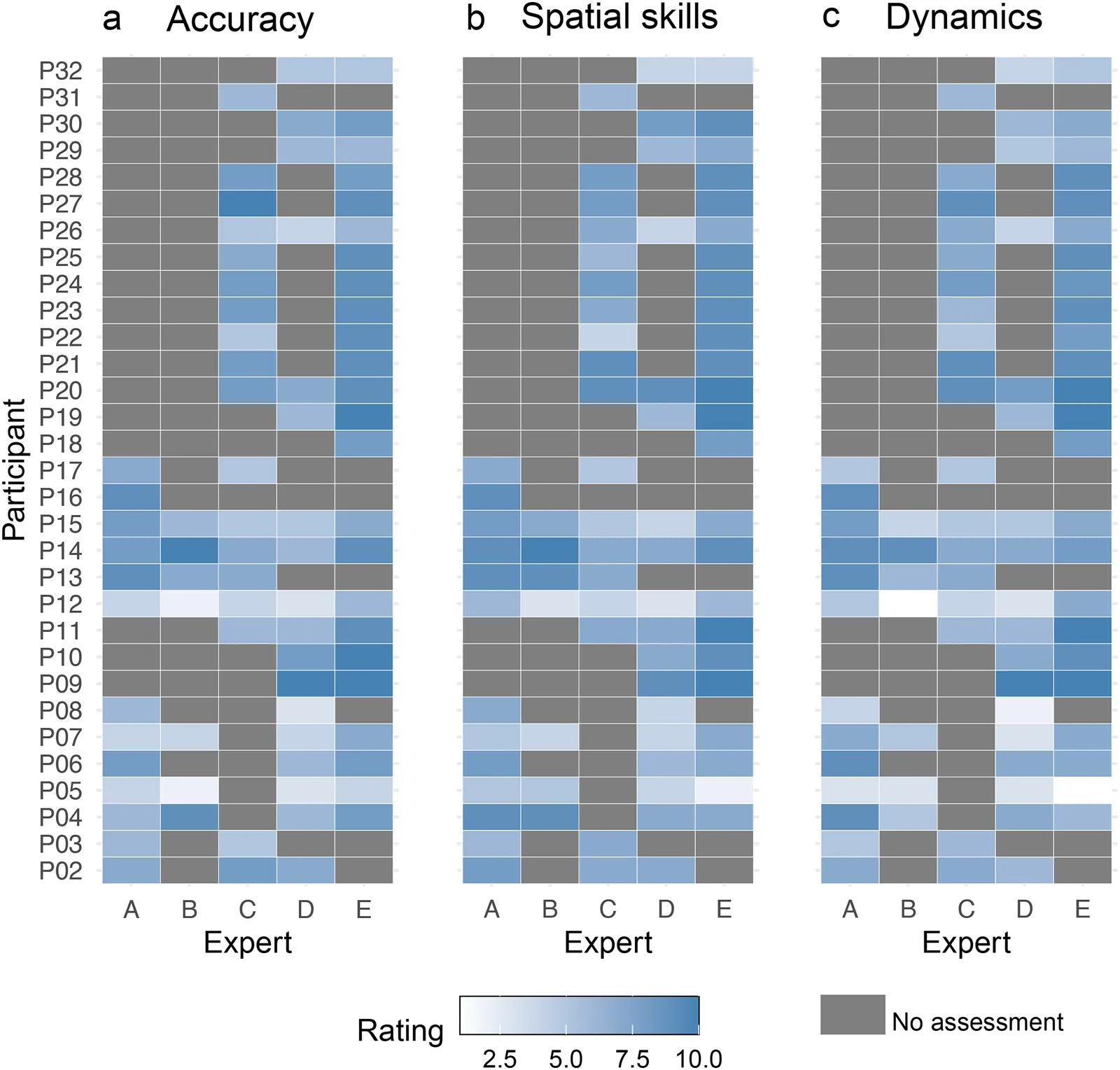
Heatmaps showing expert ratings for the participants across three performance variables: (A) Accuracy of movements, (B) Spatial skills, and (C) Dynamics and Rhythmical accuracy. Each row corresponds to a participant, each column represents one of five expert raters (A–E). Ratings range from 1 (poor) to 10 (high), with darker shades indicating higher accuracy. Grey cells indicate missing ratings. The use of a consistent colour scale across all three plots enables direct comparison between variables. Three participants (P12, P14, P15) have been rated by all experts.

**Fig 3:**
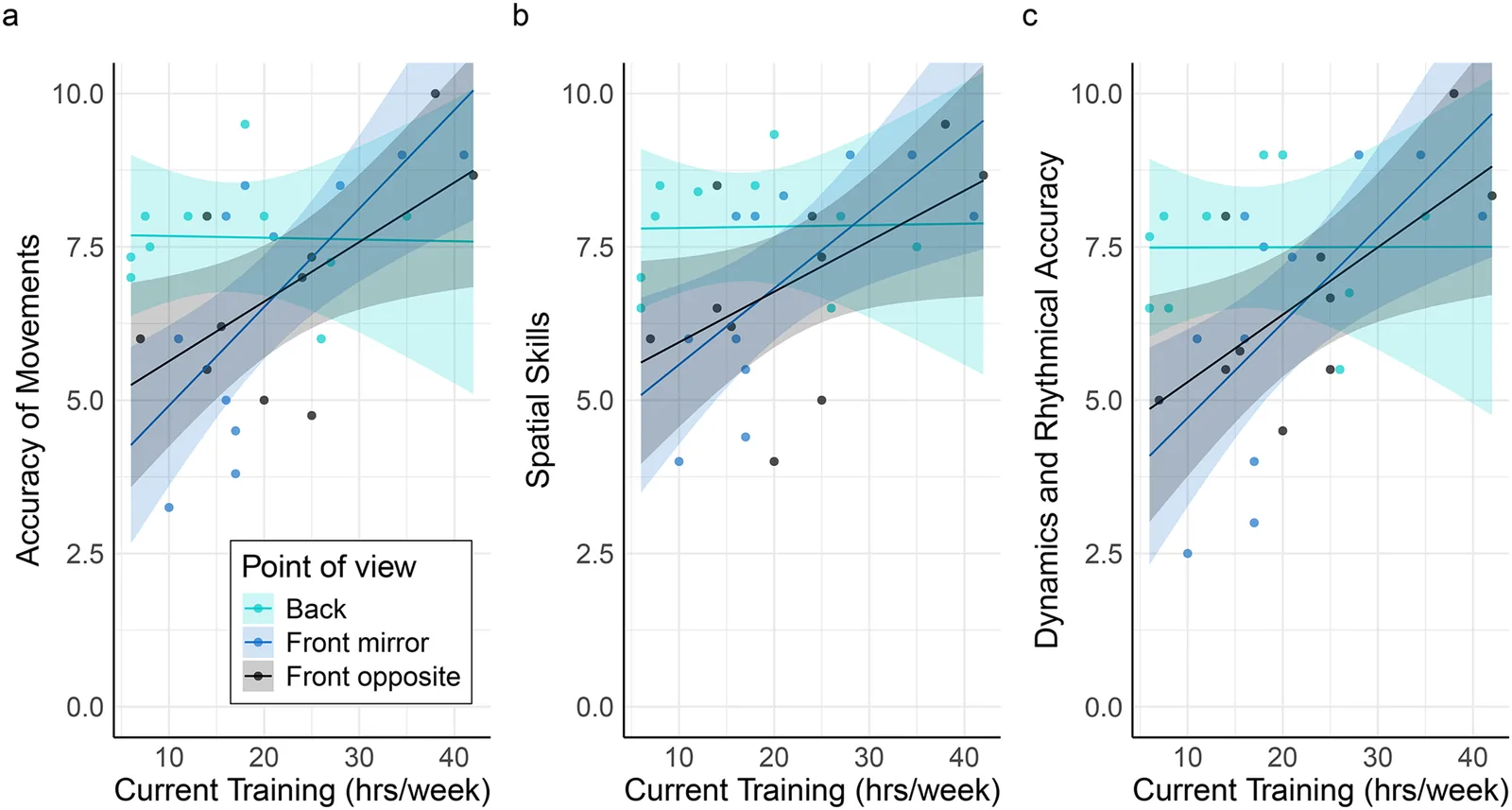
Effect of Point of View and Training on the performance, focusing on (a) Accuracy of movements, (b) Spatial skills, (c) Dynamics and rhythmical accuracy. Solid lines and error bars indicate predicted values and 95% confidence intervals respectively.

Second, the *extraneous load* refers to how the instructional material is presented (rather than to the intrinsic characteristics of the learned material), with the effect of hindering or supporting learning [28]. For instance, slower instructions enhance learning in tasks with medium and high complexity [29]. In this work, we study the effect of the extraneous load imposed by the instructional point of view. An efficient instructional design should reduce the extraneous cognitive load [30,31]. We used a between-subjects design to investigate the impact of three different teacher viewing perspectives on dance learning (see Fig 1): (1) first-person view from behind the teacher (Back view, hypothesised to impose the lowest extraneous cognitive load, Fig 1a), (2) third-person view mirrored, namely the viewpoint of someone facing the mirror image of the instructor (Front mirror view; expected to impose a moderate extraneous cognitive load, Fig 1b), and (3) third-person view without a mirror image (Front opposite: expected to impose the highest cognitive load, Fig 1c). We focused on a choreography of medium complexity, that required a minimum of three years of ballet training to be learnt in two teaching sessions of approximately 60 minutes.

Based on the cognitive load theory, our predictions were as follows: that the extraneous cognitive load is lower (and performance better) in the first-person point of view, intermediate in the third person mirrored and higher in the third person non-mirrored condition. Cognitive studies on mental rotation and mental imagery have shown longer processing times (higher cognitive load) for objects and bodies that must be “mentally rotated” at greater angular distances to perform matching tasks [32–34]. Recognition of mirror images, such as those of a teacher demonstrating a sequence in front of the learner, depends on the point of view of the observer [35]. Moreover, different studies have found a better accuracy of execution after participants had viewed an instructional video that demonstrated simple step-by-step procedures from a first-person perspective, compared to a third-person perspective [36–39]. However, a first-person perspective can also hinder dance learning, as the face and gestures of the instructor are less visible, and this is a non-canonical view, to which practitioners are less exposed.

To investigate whether learning a choreography is more effective when taught from a first-person perspective, a mirrored third-person view, or a standard third-person perspective, we used pre-recorded videos of one choreographer explaining and demonstrating a dance sequence from different points of view, as shown in Fig 1.

### Validity and applicability of the experimental setting

In this study, we investigated the cognitive load involved in acquiring a complex motor sequence by examining dancers as they learned a new solo piece of choreography. The task is directly relevant to real-life scenarios, as both in training and professional settings dancers learn new sequences to be recalled and performed later, independently. This exposes dancers to significant cognitive demands. Moreover, dancers are described as “performing” or “aesthetic athletes” [20], due to the simultaneous physical and artistic challenges of their discipline. This unique combination of demands makes dancers an ideal model for studying cognitive load and learning from instructions, with potential relevance to other contexts.

The setting based on teaching from different perspectives is ecologically valid, as in dance training and professional practice, exercises and choreographies are taught by teachers located in front of the learner, performing the same sequence from their own point of view, reversing teachers’ instructions between the right and left side, and from the third to first person points of view. For instance, from early stages of ballet training, exercises at the barre are usually taught from an ego-centric frame of reference, with all pupils starting with the left hand on the barre, irrespective of their position with respect to the teacher. Moreover, not only do dancers often learn choreography from video recordings taken from the point of view of the audience (in third person), but online instruction has become ubiquitous in professional, vocational and recreational settings since the expansion of digital platforms and social media (e.g., TikTok, Instagram, YouTube) and the transformation of teaching imposed by the COVID-19 pandemic, [2,3]. In the video formats, dancers, demonstrators and teachers are typically viewed from a third-person perspective.

Despite most dance teaching happening in the third person perspective, the work by Fiorella et al. [36] suggests that learning simple motor sequences in the first perspective from instructional videos is more effective than learning the same task in third person videos. Moreover, Koning et al. [38] showed that learning the manipulative-procedural task of knot-tying from video animations is more effective from the first-person compared to the third person perspective. Whether the same principle applies to sports and aesthetic disciplines such as dance, remains unknown.

### Aims and hypotheses

The main aim of this study was to clarify the role of the viewing perspective as a source of extraneous cognitive load. We tested the hypothesis that dance learning is enhanced when the teacher is seen in first-person perspective (Back view), compared to when they are seen in third-person perspective (Front view), as predicted by the reduced cognitive load of the first-person view. We also predicted that the third-person mirror view (Front mirror) produced a better performance compared to the third-person non-mirror view (Front opposite), as mentally rotating an image produces a higher cognitive load.

The second aim of this study was to test the effect of the expertise (intrinsic cognitive load) and its interaction with the point of view (extraneous load), in enhancing dance learning. Based on the expertise reversal effect theory, less expert practitioners were expected to benefit more from the reduction of cognitive load compared to more expert practitioners.

We also had the applied goal of measuring the reliability of our open-source digital tool MotionPerfection [40], implemented at www.wemovetogether.uk, to support the objective assessment of dance performance.

## Materials and methods

### Preregistration

The main aim of this study was preregistered on AsPredicted.org prior to data collection (Registration number: 66,583, 22 May 2021). The preregistration included hypotheses, study design, and planned analyses.

### Participants

After ethical approval from the Queen Mary University Research Ethics Committee (QMREC2020/34), we recruited voluntary participants among active dancers aged 18 years or older (range 18–43 years old, M = 23, SD = 5.15), with at least 3 years of ballet training. Dancers were recruited using a convenience sampling approach via advertisements at three London-based vocational dance schools and through affiliated dance teachers. Recruitment started on June 2021 and ended on January 2023. We recruited a total of 32 participants (27 females and 5 males). One participant was excluded due to a procedural error (incorrect point of view presentation), resulting in a final analysed sample of 31 participants (26 females, 5 males). Participants were drawn from three different backgrounds: vocational ballet schools (n = 4), vocational contemporary dance schools (n = 8), and independent dancers not enrolled in vocational training (n = 19). Participants were stratified by sex and randomly assigned to different experimental conditions.

The pre-registered design targeted a relatively homogeneous sample of highly trained dancers, with an expected small effect size (f = 0.26; α = 0.05, power = 0.8; target N ≤ 147). As shortly before data collection lockdown restrictions were lifted and in-person teaching resumed, recruitment opportunities were substantially reduced for the vocational students initially targeted. To address this challenge, we broadened recruitment to include dancers with a minimum experience of three years of ballet, which was needed to match the requirements of the choreography. While this introduced greater variability in participants’ expertise, it increased the expected effect size and allowed us to explore a key aspect of cognitive load theory: the differences in learning between different levels of expertise.

### Experimental design

In this confirmatory pre-registered study, we investigated the effect of cognitive load imposed by observing the dance teacher from different points of view, testing participants in three experimental conditions (see Fig 1): first-person view: Back (lowest expected cognitive load), third person view: Front matched (intermediate expected cognitive load), and third person view: Front opposite (highest expected cognitive load). Cognitive load was not directly measured; instead, we interpreted differences in performance across conditions as reflecting variation in processing demands induced by the instructional perspective. The use of primary task performance to infer cognitive load dynamics is validated methodology [31,41,42].

To assess dancers’ performance, dance experts evaluated: Control of movements; Accuracy of movements; Technique; Spatial Skills; Dynamics and Rhythmical accuracy; Performance qualities. Assessment criteria, adapted from Angioi et al. [20] are summarised in S1 Table. Dance experts also assessed the proportion of errors in body segment positions (matching of joint positions between a model and individual dancers, in 13 poses). As this variable did not allow discrimination between participants, and did not capture the complexity of dance dynamics and artistry, we focused on the other measures of performance. Because dancers came from different training backgrounds, this was accounted for by using as covariate the current training expertise of dancers, based on the weekly hours of dance and physical training at the time of the experiment.

### Apparatus

The dancers watched the recording of the teacher from a monitor connected to a laptop, during a recorded Zoom call. The dance floor was a square, 2 m side, delimited by coloured tape. Experimenters helped the dancer to setup the square making sure that dancers were fully visible throughout the recording. To support and systematise the assessment, scoring and annotation, the dance experts assessed dancers’ performance using the digital platform www.wemovetogether.org, that implements our open access framework [40].

### Stimuli and test

Participants were randomly assigned to three conditions: either Back (for participants to copy), Front mirror (for participants to match the image seen, as in a mirror) or Front opposite (for participants to flip the steps, as in an interaction with a teacher located in front), see Fig 1.

Conditions differed only in the point of view of the teaching recording. To make sure that participants assigned to the different conditions had the same instructional information, we video recorded the choreographer simultaneously from the front and back points of view, while he provided verbal and visual instructions. The teacher (Mr Erico Montes) was an expert ballet master, ballet teacher and choreographer, former professional dancer at the Royal Ballet, with hundreds of hours of teaching experience. To obtain the Front mirror video, we flipped the left/right side of the Front opposite image. The same audio file was used for all three videos. Videos were recorded using a GoPro Hero 7 camera.

The choreography lasted 1’15” overall. In the first session, the entire choreography was taught. In the second and last session, the instructor rehearsed the choreography, to prepare dancers for the performance. To reduce the risk of injury, the choreographer included no high impact jumps and only a single turn [43,44]. In line with this, no injuries or physical issues have been recorded during the experiment.

### Procedure

All participants took part in two sessions, on two consecutive days. After recruitment, participants were given an information sheet, had the opportunity to ask questions, and then signed the consent form. We recorded demographic information before Session 1, and feedback on the project at the end of Session 2.

In Session 1, participants completed the demographic questionnaires and then proceeded to the video-recorded session. The video-recorded session included a 20-minute warm-up, followed by a 60-minute choreographic session led by the choreographer. To familiarise participants with the performance format, Session 1 ended with two solo attempts, where participants performed the choreography without any external feedback. In Session 2, after the warm-up and a 45-minute rehearsal session, participants performed the choreography twice, without any external feedback. We assessed performance using the second recording.

### Video analysis

Objective assessment is a key challenge for aesthetic disciplines such as dance [45]. To support and standardise this task, dance experts conducted structured video-based assessments on the freely available online platform https://www.wemovetogether.uk/, based on the open source software MotionPerfection [40]. The software is designed for the visualisation, synchronisation and annotation of videos that display motor sequences with an acoustic component to anchor the execution time.

Dance experts assessed the execution using the Aesthetic Competence Tool [20], which is based on audition criteria of international dance schools and dance companies across the United Kingdom, Australia and USA. Our analyses focused on the three criteria of the Aesthetic Competence Tool that correspond to our pre-registered measures of interest: Accuracy of movement, Spatial skills, Dynamics/timing/rhythmical accuracy. Dance experts scored dancers for all the seven criteria of the scale, and we observed (as an exploratory analysis) that all the measures (including also Control of movement, Technique; Performance qualities and Overall performance) exhibited the same pattern of the pre-registered variable of interest.

Participants’ performance was assessed by five dance experts: four with direct experience in vocational training and dance performance, one with dance training. Dance experts were blind to the experimental condition and level of training of each participant. Each participant was assessed by at least two experts, except for Participant 16, 18, 31, who were assessed by one dance expert only, due to limited availability of the experts.

Each performance variable was rated on a scale from 10 to 0, where 10 was the best possible execution, 1 an extremely poor execution and 0 no execution. All dancers executed the choreography. Experts also assessed the individual pose accuracy of body segment position, focusing on 13 core poses compared to the position held by the choreographer. As this approach was not sensitive enough to participant differences, due to the low number of errors, we focused the analyses on the established artistic elements of choreography.

Dance experts further quantified the number of errors looking at 13 static poses during the choreography, for which the teacher explicitly trained the participants, comparing the static executed pose, with that of a “ground-truth” model. Due to the low number of errors in the static poses and to the dynamic nature of dance, this measure was not sensitive enough. Moreover, all dance experts commented that the evaluation of poses was not adequate to capture the core of dance performance. Based on this, data analyses focused on the Aesthetic Competence Tool results only.

### Data analysis

Analyses and plots were performed conducted in R (R version 4.4.2 (2024-10-31) via R-studio 2023.12.1.402). Data are available in the S1 File. For all analyses we set a significance level of p ≤ 0.05.

We initially assessed the inter-rater reliability using Intraclass Correlation Coefficients (ICC) [46,47], as visualised in Fig 2. We first examined a subset of three participants (P12, P14, P15) who had been rated by all five experts. This subset analysis revealed substantial agreement among individual raters (ICC[2]=0.66) and excellent reliability when averaging across raters (ICC[2k]=0.91). While based on a small sample (n = 3), this initial result demonstrated strong consistency when complete rater coverage was present. To validate these findings more broadly, we then assessed inter-rater reliability across the full sample of participants, including those with some missing ratings. The ICC analysis showed moderate to substantial agreement among individual raters (ICC[1]=0.58, ICC[2]=0.59, ICC[3]=0.69), and excellent reliability when averaging scores (ICC[1k]=0.87, ICC[2k]=0.88, ICC[3k]=0.92). These results support the use of averaged expert ratings as a reliable measure of movement accuracy in this context.

Before inferential analyses, we examined the interrelationships among the dependent variables Accuracy of movements, Spatial skills, Dynamics and Rhythmical accuracy. Pearson correlation analysis showed high correlations among all three variables (r ≈ 0.90). This strong multicollinearity likely reflects a common underlying construct in performance. For completeness, we report results of all three variables, as they are potentially independent (e.g., accurate movements can be performed in the wrong spatial location, at the wrong time). As these measures are often used together in dance assessments, it will be informative to provide full reporting of all metrics to facilitate future meta-analyses and establish standardized benchmarks in dance science.

To examine whether dancers’ viewing perspective of the teacher (Point of View: Back, Front mirror, Front opposite), expertise (measured as weekly hours of training), and their interaction influenced dance performance, we fitted a linear model with the average expert ratings as a continuous dependent variable (Accuracy of movements, Spatial skills, Dynamics and rhythmical accuracy). To further characterise the interaction between Training and Point of View, we used post hoc analyses using the emmeans package. Simple slopes of Training (weekly hours) were estimated separately for each Point of View condition. Differences between slopes were assessed using pairwise comparisons with Tukey adjustment for multiple testing. This approach allowed us to determine whether the relationship between Training and performance differed across viewing perspectives.

For all dependent variables, model diagnostics indicated good fit of the linear regression model. Residual plots showed no obvious deviations from homoscedasticity or normality, and the Shapiro-Wilk test confirmed normality of residuals. No influential points were detected via Cook’s distance. To visualise the results, we computed predicted values using the *ggeffects* packag*e* [48]*,* which calculates marginal effects (predicted means) from the fitted model, while accounting for interactions. Specifically, we used the function *ggpredict* to generate predicted values for the dependent variables across a range of current training hours, for each video condition. Predicted values, including 95% confidence intervals, were plotted alongside the raw participant data using the *ggplot2* package [49].

### Deviations from pre-registration

#### Sample size and sample composition.

This study was originally planned and funded as an acute response to the lockdown training experienced during the Covid-19 pandemic, focusing on isolated individual vocational dance students (16–18 years), with a similar level of training. However, as the lockdown was lifted, vocational dancers were extremely busy with in-person group classes, so that only 4 vocational dancers could enrol in this experiment. Hence, we have been forced to reduce the sample size and to open-up registration to non-vocational dancers. As inclusion criteria, we required participants to be currently active in dance and to have at least 3 years of experience in ballet. Because we included dancers with levels and type of experience, we used training practice as a proxy for expertise. We measured training as the number of hours spent in physical training every week. Accounting for expertise is essential, as it can dramatically affect the cognitive load (see Introduction).

#### Dependent variables.

As per preregistration protocol, dance experts scored individual dancers’ performance. In the preregistration we planned to analyse the proportion of errors (number of errors/overall visible positions or segments) for each dancer at predefined times as dependent variable, focusing on (a) errors in the position of body segments with respect to the own body, (b) errors in direction in space and (c) timing errors at 20 specific time points. However, due to the need to use a short choreography that could fit a 2-day project, and to the small number of errors detected with the analysis of static poses, this approach was not sensitive enough. Hence, we asked dance experts to assess the dancers’ overall performance, focusing on the established measures of Accuracy of movements, Spatial skills, Dynamics and Rhythmic accuracy [20].

## Results

### Performance

#### Accuracy of movements.

To examine the effect of Point of View and Training on accuracy of movements, we fitted a linear model including main effects and their interaction (Fig 3a). The overall model was significant, F_5,25_ = 4.33, p = 0.006, explaining 36% of the variance (adjusted R² = 0.36). At the effect level, we observed a significant main effect of Training (F_1,25_ = 11.463, p = 0.002), Point of View (F_2,25_ = 6.093, p = 0.007) and their interaction (F_2,25_ = 3.459, p = 0.047). At the coefficient level (Table 1), relative to the Front opposite condition, the Back condition showed higher accuracy (β = 2.48, p = 0.003), whereas the Front mirror condition showed lower accuracy (β = −1.92, p = 0.026). Training level was positively associated with accuracy (β = 0.085, p = 0.002). These coefficients are consistent with the observed interaction.

**Table 1:** Summary of the linear regression model examining the effects of *Training* (weekly hours), *Point of View* (Back, Front mirror; reference category: Front opposite), and their interaction on *Accuracy of movements*. The table reports model estimates, standard error of the mean, t-values, and p-values for each predictor.

	Estimate	Std. Error	t value	p
Intercept	5.224	0.559	9.342	<0.001
Training	0.085	0.025	3.386	0.002
Back	2.481	0.744	3.334	0.003
Front mirror	−1.921	0.810	−2.371	0.026
Training*Back	−0.088	0.036	−2.452	0.022
Training*Front mirror	0.076	0.036	2.107	0.045

To further characterise the interaction between Training and Point of View, we examined simple slopes of Training for each condition. Training was not reliably associated with accuracy in the Back condition (β = −0.003, SE = 0.044, 95% CI [−0.094, 0.088]), whereas it was positively associated with accuracy in both the Front mirror condition (β = 0.161, SE = 0.045, 95% CI [0.069, 0.252]) and the Front opposite condition (β = 0.097, SE = 0.042, 95% CI [0.011, 0.183]). Pairwise comparisons of these slopes indicated that the effect of Training was significantly stronger in the Front mirror condition than in the Back condition (Δβ = 0.164, SE = 0.063, p = 0.039, Tukey-adjusted). Differences between the Back and Front opposite conditions (p = 0.246) and between the two Front conditions (p = 0.558) were not statistically significant. The full table of coefficients is shown in Table 1.

#### Spatial skills.

To examine the effect of Point of View and Training on spatial skills, we fitted a linear model including main effects and their interaction (Fig 3b). The overall model was significant, F_5,25_ = 2.95, p = 0.032, explaining 24% of the variance (adjusted R² = 0.24). At the effect level, we observed a significant main effect of Training (F_1,25_ = 7.82, p = 0.010) and Point of View (F_2,25_ = 3.97, p = 0.032), but their interaction was not significant (F_2,25_ = 1.99, p = 0.158).

At the coefficient level (Table 2), relative to the Front opposite condition, the Back condition showed higher spatial skills (β = 2.04, p = 0.010), whereas the Front mirror condition did not differ significantly (β = −1.41, p = 0.092). Training level was positively associated with spatial skills (β = 0.070, p = 0.010). The interaction terms were not significant (although showed the same trends observed for accuracy of movement), indicating that the effect of Training did not differ reliably across perspectives. The full table of coefficients is shown in Table 2.

**Table 2:** Summary of the linear regression model examining the effects of *Training* (weekly hours), *Point of View* (Back, Front mirror; reference category: Front opposite), and their interaction on *Spatial skills*. The table reports model estimates, standard error of the mean, t-values, and p-values for each predictor.

	Estimate	Std. Error	t value	p
Intercept	5.746	0.555	10.348	<0.001
Training	0.070	0.025	2.797	0.010
Back	2.039	0.744	2.759	0.010
Front mirror	−1.411	0.804	−1.754	0.092
Training*Back	−0.067	0.036	−1.894	0.070
Training*Front mirror	0.054	0.036	1.533	0.138

The main effects indicate that greater levels of training were associated with higher spatial skill scores, and that performance varied depending on the viewing perspective. Estimated marginal means showed that participants in the Back view condition had the highest spatial skills scores, while those in the Front views (Mirror and Opposite) scored lower on average. Although the pairwise comparisons were not significant, effect size estimates suggested a moderate advantage for the Back view over both Front perspectives: Cohen’s d ≈ 0.75–0.79 for the Back vs Front conditions, whereas Cohen’s d ≈ 0.040 for the Front mirror vs Front opposite difference. Given that these estimates were associated with non-significant comparisons, they should be interpreted cautiously, as do not provide conclusive evidence for differences between conditions.

#### Dynamics and rhythmical accuracy.

To examine the effect of Point of View and Training on dynamics and rhythmic accuracy, we fitted a linear model including main effects and their interaction (Fig 3c). The overall model was significant, F_5,25_ = 5.05, p = 0.003, explaining 38% of the variance (adjusted R² = 0.38). At the effect level, we observed a significant main effect of Training (F_1,25_ = 10.17, p = 0.004) and Point of View (F_2,25_ = 4.99, p = 0.015). There was also a trend toward an Interaction between Training and Point of View (F_2,25_ = 2.65, p = 0.091). At the coefficient level (Table 3), relative to the Front opposite condition, the Back condition showed higher dynamics and rhythmical accuracy (β = 2.54, p = 0.005), whereas the Front mirror condition did not differ significantly (β = −1.79, p = 0.057). Training level was positively associated with performance (β = 0.088, p = 0.004). The full table of coefficients is shown in Table 3.

**Table 3:** Summary of the linear regression model examining the effects of *Training* (weekly hours), *Point of View* (Back, Front mirror; reference category: Front opposite), and their interaction on *Dynamics and rhythmical accuracy*. The table reports model estimates, standard error of the mean, t-values, and p-values for each predictor.

	Estimate	Std. Error	t value	p
Intercept	4.95	0.618	8.012	<0.001
Training	0.088	0.028	3.189	0.004
Back	2.537	0.822	3.087	0.005
Front mirror	−1.786	0.894	−1.997	0.057
Training*Back	−0.088	0.039	−2.224	0.035
Training*Front mirror	0.066	0.040	1.677	0.106

The trend in the interaction suggests that the relationship between training and performance may vary by viewing perspective. Participants in the Back view condition showed relatively high rhythmic accuracy even at lower levels of training, whereas participants in the Front views appeared to benefit more clearly from additional training. Effect size estimates supported these differences, with moderate to large advantages for the Back view over both Front perspectives (Cohen’s d ≈ 0.73–0.82). Although confidence intervals for these effect sizes included zero, indicating these differences were not statistically significant at the 0.05 level, the pattern of results suggests a practical advantage for the Back view condition in dynamics and rhythmical accuracy. Conversely, the effect size of difference between the two Front views was negligible (Cohen’s d = −0.089).

## Discussion

We investigated how different visual perspectives of a dance instructor (first-person Back view, third-person Front mirror, and third-person Front opposite) influence dance learning and performance, as moderated by participants’ training experience. This addresses the issue of extraneous cognitive load (cognitive load dependent on the instructional layout) in motor learning. Our pre-registered hypothesis predicted that the Back view would support better learning outcomes by imposing the lowest extraneous cognitive load. The Back view in fact corresponds to a first-person visual alignment of the dancer with the instructor and the exercise. As more expert dancers have lower cognitive load for a given level of choreographic complexity, the largest advantages were expected in less experienced dancers. We also predicted an intermediate load and performance for the Front mirror view, namely a third person perspective in which the learners move on the same direction of the demonstrator, and a heavier load for the Front opposite view, in which the learner moved in opposite direction of the demonstrator.

Overall, this pattern is consistent with the hypothesis of the lower cognitive load for the Back view, as we observed an enhanced performance either as a significant effect or as a trend across all variables: accuracy of movements, spatial skills, and dynamics/rhythm. Front views posed greater challenges, particularly for dancers with lower training levels. The data did not provide full support for the predicted differentiation between the two front conditions, as performance was not significantly different between Front mirror and Front opposite views. This suggests that the primary distinction captured in this study may be between body-aligned (Back) and front-facing perspectives, rather than between graded levels of front-view difficulty. However, the lack of significant differences between the two front views could also be attributed to the reduced sample size, which was impacted by the recruitment challenges following the ease of the COVID-19 restrictions, and that forced us to reduce the recruitment targets. Indeed, a limitation of our study is the reduced sample size, as recruiting dancers specifically trained in ballet for individual sessions across multiple days has been a challenge. Hence, one possibility is that limited statistical power or participants’ prior exposure to both front-facing perspectives reduced sensitivity to detect differences between these conditions. Alternatively, or additionally, the lack of significant differences between mirror and opposite front views can reflect the current exposure of dancers to both perspectives in their in-person dance training, or to the widespread exposure to third-perspective videos via social media and digital tools widely available. Future studies should clarify how different types of training affect cognitive load longitudinally.

The observed advantage for the Back view aligns with evidence that “mental rotation” and spatial transformations increase cognitive load and hinder performance [32–35]. In our case, the Back view appears to mitigate this load, by allowing participants to map observed movements directly onto their own experience, producing a better performance. Our findings are also consistent with studies in basic procedural learning via video instruction, where first-person demonstrations enhance learning [36,38]. Our findings suggest that the lack of direct experience with the face and gestures of the instructor as seen mostly from the back, does not substantially hinder learning under these conditions.

Confirming other research (e.g., 27), our findings showed that training experience significantly affected performance, with more experienced dancers performing better than less experienced ones. The performance gap due to experience was especially evident in dancers with fewer than 25 hours of weekly training, the only group to receive scores in the lower range.

The effect of Point of View and Training is better understood when considering their interaction, at least for the movement accuracy. An interaction between Point of View and Training was observed for movement accuracy, whereas this pattern was weaker or not statistically robust for the other outcomes. The modulation by expertise was clearest for movement accuracy. In the contest of movement accuracy, the effect of the point of view was strongly affected by the expertise of dancers, as shown by the significant interactions on Point of View and Training. This suggests that, in front of the same choreography, different strategies might be needed for dancers with different levels of expertise. While the fact that the Back view appeared to particularly facilitate less experienced dancers suggests that the benefit of a first-person perspective is higher in less experienced dancers, only few experienced dancers have been tested on the Back point of view. Taken together, these findings provide some partial support for an expertise-reversal account.

These results pave the way to further studies on the effectiveness of the first-person perspective for experienced dancers with challenging choreographies, and whether the benefit of first-person point of view recapitulates the expertise reversal effect [24]. Future studies should clarify to what extent expert professional dancers, who are often requested to learn a choreography from videos, could benefit from a first-person rather than third person perspective. Taken together, our results suggest that first-person perspectives may represent a useful strategy to adapt instructions to learner expertise and facilitate the task. The first-person view is often under-utilised in real-world dance education, in particular in ballet classes, where dancers training at the barre are often asked to start the exercise on the same egocentric position (e.g., usually with the left hand on the barre), irrespectively of whether it is the same or the different side of the demonstrator/teacher in front of them [50]. Our study opens the way for further investigation on the efficacy of first-person perspective in learning a choreography or motor sequences with different levels of expertise and developmental stages.

The potential impact of dance and motor sequence learning enhancement is huge, as dance is a behaviour observed in all human cultures [51,52]. For instance, according to the official data of the UK [53], in this country there are approximately 7,000 dancers and choreographers who undergo daily training and are engaged in the teaching and learning choreographies through in person or video instruction. In England, 27% of children and young adults take part at least once a week to recreational dance classes in person or online, a format increasingly popular since the COVID-19. Other disciplines directly rely on choreographies and complex motion sequences from artistic and rhythmic gymnastics to figure skating, synchronised swimming, but the teaching and learning of motor routines is a broader issue across sports.

Overall, our findings indicate that a first-person perspective may support motor sequence learning, when high cognitive load is involved. By confirm high inter-rater reliability in performance, we validated a digital tool for the analysis and assessment of motor sequences. These outcomes may have theoretical and practical implications for the advancement of instructional design, the practice and teaching of dance. Future studies should assess the generalisability to other disciplines in performing arts and sports that require complex sequence learning, both in video/online and in-person settings. On the one hand, as remote learning and video-based teaching become increasingly common, instructional design should consider the cognitive impact of camera angle and perspective. Conversely, as dance training typically engages practitioners of different expertise with potentially challenging third-person perspectives, teachers and choreographers should consider shifting their position, or that of their students, to overcome difficulties of less experienced athletes. This may support motor sequence learning in dance and beyond.

## Supporting information

S1 Table Assessment criteria and scoring scale. (DOCX)

S1 File Dataset: Support dataset. (TXT)
