# Supplementary material for: Enhancing dance learning by optimising instructional perspective: A preregistered study on the effect of dancers’ viewing perspective, cognitive load and expertise
Source: PLoS One. 2026 Sep 16;21(9):e0355860. doi: 10.1371/journal.pone.0355860 (PMC13581001; doi:10.1371/journal.pone.0355860)
Supplement: Supplementary file 1 [file pone.0355860.s001.docx]

**Supplementary 1 Table. Assessment criteria and Scoring scale**

| **Criteria** | **Scoring scale** |
| --- | --- |
| **Control of Movements**  Controlled landing from jump/turn, controlled lifting/lowering of limbs, controlled shifting of body weight. Core strength, alignment, posture. | (empty): Not applicable  0: Not executed  1–3: Some evidence of coordination, movement control, and body awareness, but limited and inconsistent.  4–6: Some elements were stronger than others.  7–8: Secure general coordination and body alignment; generally well-controlled movements.  9–10: Well-coordinated movement and controlled work all of the time, with accurate alignment. |
| **Spatial Skills**  Spatial awareness, accuracy and intention. | (empty): Not applicable  0: Not executed  1–3: Little or no use of peripheral space; poor use of performance space.  4–6: Some good use of space, but inconsistent. Some elements stronger than others.  7–8: Good use of space about 80% of the time, with general accuracy and intent.  9–10: Secure and confident use of space, with accuracy and intent. |
| **Accuracy of Movements**  Arm placement, feet positions, fully stretched leg extensions (if required). | (empty): Not applicable  0: Not executed  1–3: Little or no precision throughout sequence. Unclear leg/arm lines.  4–6: Some precision, but inconsistent. Some elements stronger than others.  7–8: Correct positioning about 80% of the time.  9–10: Precise placing with well-articulated gestures of limbs. |
| **Technique**  Elevation, turning and jumping techniques, height of extensions, balance, posture, placement, articulation. | (empty): Not applicable  0: Not executed  1–3: Little or no evidence of high technical skill in any element.  4–6: Some skill in some elements, general virtuosity achieved.  7–8: Good virtuosity shown about 80% of the time.  9–10: A stunning performance showing virtuosity and skill throughout. |
| **Dynamics and Rhythmical Accuracy**  Dancing with correct timing and ability to perceive movement and rhythmic patterns. Showing awareness of changes in musical dynamics and phrasing. | (empty): Not applicable  0: Not executed  1–3: Little or no ability to perform and respond in time to the music. Little or no dynamic qualities.  4–6: Performed in time for over half of the sequence, with some ability to respond to different rhythms and dynamics.  7–8: Timing accurate for most of the sequence; good use of dynamics; good sense of musicality.  9–10: Timing accurate throughout, with excellent response to rhythms, dynamics, and phrasing. |
| **Performance Qualities**  Ability to execute the work for an audience. Presence, expressiveness, memory recall. | (empty): Not applicable  0: Not executed  1–3: Few or no performance qualities shown. Poor memory recall.  4–6: Some performance qualities shown. Generally good memory recall.  7–8: Strong expressive qualities and memory recall about 80% of the time.  9–10: Excellent projection of expression and emotion; mature performance. |
| **Overall Performance**  Does the performance overall impress markers? | (empty): Not applicable  0: Not executed  1–3: Dancer made little impression on the audience.  4–6: Dancer not at full potential yet, OR strong work but lacking ability to impress overall.  7–8: Dancer has ability to shine, but hindered by minor aspects.  9–10: Impressive! |
