# Supplementary material for: Enhancing dance learning by optimising instructional perspective: A preregistered study on the effect of dancers’ viewing perspective, cognitive load and expertise
Source: PLoS One. 2026 Sep 16;21(9):e0355860. doi: 10.1371/journal.pone.0355860 (PMC13581001; doi:10.1371/journal.pone.0355860)
Supplement: Supplementary file 2 [file pone.0355860.s004.docx]

Response Reviewers PLOS

PLOS ONE Decision: Revision required [PONE-D-26-00870] - [EMID:85ac42c087f8629b]

[em.pone.0.9ae5e6.11209e71@editorialmanager.com](mailto:em.pone.0.9ae5e6.11209e71@editorialmanager.com) on behalf of PLOS ONE

Elisabetta Versace

PONE-D-26-00870

Enhancing dance learning by reducing cognitive load: A preregistered study on the effect of dancers’ viewing perspective and expertise

PLOS One

Dear Dr. Versace,

Thank you for submitting your manuscript to PLOS ONE. After careful consideration, we feel that it has merit but does not fully meet PLOS ONE’s publication criteria as it currently stands. Therefore, we invite you to submit a revised version of the manuscript that addresses the points raised during the review process.

==============================
**ACADEMIC EDITOR**

Dear Authors,

Your manuscript has been reviewed by an expert in the field. During the review process, several critical issues emerged that should be carefully addressed in the revision phase.

Dear Editor,

Thank you for the opportunity to revise and strengthen our manuscript. We greatly appreciate the constructive feedback, which has helped us enhance both the clarity and scientific robustness of our paper.

We carefully considered all suggestions and addressed all the comments by the editors and reviewers point by point below. The manuscript has been updated accordingly. More specifically, we have 1) clarified our analyses and 2) included more direct comparisons to better unpack the significant interaction, as requested. Furthermore, we have removed any potential overstatements and refined our interpretations to ensure our conclusions are appropriately grounded, as suggested by the reviewers.

We are confident that these revisions have significantly strengthened the paper, and we hope it is now suitable for publication.

Sincerely,

Elisabetta Versace and Manuela Angioi on behalf of all authors

==============================

Please include the following items when submitting your revised manuscript:

- A letter that responds to each point raised by the academic editor and reviewer(s). You should upload this letter as a separate file labeled **'Response to Reviewers'**.

Done

- A marked-up copy of your manuscript that highlights changes made to the original version. You should upload this as a separate file labeled **'Revised Manuscript with Track Changes**'.

Done

- An unmarked version of your revised paper without tracked changes. You should upload this as a separate file labeled '**Manuscript**'.

Done

If you would like to make changes to your financial disclosure, please include your updated statement in your cover letter. Guidelines for resubmitting your figure files are available below the reviewer comments at the end of this letter. If applicable, we recommend that you deposit your laboratory protocols in protocols.io to enhance the reproducibility of your results. Protocols.io assigns your protocol its own identifier (DOI) so that it can be cited independently in the future. For instructions see: [https://journals.plos.org/plosone/s/submission-guidelines#loc-laboratory-protocols](https://eur01.safelinks.protection.outlook.com/?url=https%3A%2F%2Ftrack.editorialmanager.com%2FCL0%2Fhttps%3A%252F%252Fjournals.plos.org%252Fplosone%252Fs%252Fsubmission-guidelines%2523loc-laboratory-protocols%2F1%2F010f019dae1529c3-baf455ed-87d7-4be6-a0b2-a1500dd0507f-000000%2F31Fj3iVdbcNhGtiuGDVnfOUroNlRg82wUmZ2c37aUc8%3D258&data=05%7C02%7Ce.versace%40qmul.ac.uk%7C9a1139c9f29e4cc918d308de9f56133c%7C569df091b01340e386eebd9cb9e25814%7C0%7C0%7C639123389671977425%7CUnknown%7CTWFpbGZsb3d8eyJFbXB0eU1hcGkiOnRydWUsIlYiOiIwLjAuMDAwMCIsIlAiOiJXaW4zMiIsIkFOIjoiTWFpbCIsIldUIjoyfQ%3D%3D%7C0%7C%7C%7C&sdata=3yjN%2B8fkxiaucavhvX%2FS3BS3HNAZY96cbEjkaSOdj0Q%3D&reserved=0). Additionally, PLOS ONE offers an option for publishing peer-reviewed Lab Protocol articles, which describe protocols hosted on protocols.io. Read more information on sharing protocols at [https://plos.org/protocols?utm_medium=editorial-email&utm_source=authorletters&utm_campaign=protocols](https://eur01.safelinks.protection.outlook.com/?url=https%3A%2F%2Ftrack.editorialmanager.com%2FCL0%2Fhttps%3A%252F%252Fplos.org%252Fprotocols%253Futm_medium%3Deditorial-email%2526utm_source%3Dauthorletters%2526utm_campaign%3Dprotocols%2F1%2F010f019dae1529c3-baf455ed-87d7-4be6-a0b2-a1500dd0507f-000000%2FJciGHJXAVDwgZjW7g1pvMwb0N2qgV0FKkMKejoTjTyw%3D258&data=05%7C02%7Ce.versace%40qmul.ac.uk%7C9a1139c9f29e4cc918d308de9f56133c%7C569df091b01340e386eebd9cb9e25814%7C0%7C0%7C639123389671992201%7CUnknown%7CTWFpbGZsb3d8eyJFbXB0eU1hcGkiOnRydWUsIlYiOiIwLjAuMDAwMCIsIlAiOiJXaW4zMiIsIkFOIjoiTWFpbCIsIldUIjoyfQ%3D%3D%7C0%7C%7C%7C&sdata=EVRoiwMFCtW2AlxnT6h7RVD6b4Ee26HFR8yv%2BM%2BdSF8%3D&reserved=0).

As the corresponding author, your ORCID iD is verified in the submission system and will appear in the published article. PLOS supports the use of ORCID, and we encourage all coauthors to register for an ORCID iD and use it as well. Please encourage your coauthors to verify their ORCID iD within the submission system before final acceptance, as unverified ORCID iDs will not appear in the published article. *Only* the individual author can complete the verification step; PLOS staff *cannot* verify ORCID iDs on behalf of authors.

We look forward to receiving your revised manuscript.

Kind regards,

Emiliano Cè, Ph.D.
Academic Editor
PLOS One

Journal Requirements:

When submitting your revision, we need you to address these additional requirements.
1. Please ensure that your manuscript meets PLOS ONE's style requirements, including those for file naming. The PLOS ONE style templates can be found at
[https://journals.plos.org/plosone/s/file?id=wjVg/PLOSOne_formatting_sample_main_body.pdf](https://eur01.safelinks.protection.outlook.com/?url=https%3A%2F%2Ftrack.editorialmanager.com%2FCL0%2Fhttps%3A%252F%252Fjournals.plos.org%252Fplosone%252Fs%252Ffile%253Fid%3DwjVg%252FPLOSOne_formatting_sample_main_body.pdf%2F1%2F010f019dae1529c3-baf455ed-87d7-4be6-a0b2-a1500dd0507f-000000%2FQQ_6zWrZJiU1mkLM_BdngOJ9Wq0fFcmKeYNyfqfhs70%3D258&data=05%7C02%7Ce.versace%40qmul.ac.uk%7C9a1139c9f29e4cc918d308de9f56133c%7C569df091b01340e386eebd9cb9e25814%7C0%7C0%7C639123389672000866%7CUnknown%7CTWFpbGZsb3d8eyJFbXB0eU1hcGkiOnRydWUsIlYiOiIwLjAuMDAwMCIsIlAiOiJXaW4zMiIsIkFOIjoiTWFpbCIsIldUIjoyfQ%3D%3D%7C0%7C%7C%7C&sdata=lZPwNRAOdcWFPhfBj5A5znJYnLzWidYiY13yCbYuM3c%3D&reserved=0) and [https://journals.plos.org/plosone/s/file?id=ba62/PLOSOne_formatting_sample_title_authors_affiliations.pdf](https://eur01.safelinks.protection.outlook.com/?url=https%3A%2F%2Ftrack.editorialmanager.com%2FCL0%2Fhttps%3A%252F%252Fjournals.plos.org%252Fplosone%252Fs%252Ffile%253Fid%3Dba62%252FPLOSOne_formatting_sample_title_authors_affiliations.pdf%2F1%2F010f019dae1529c3-baf455ed-87d7-4be6-a0b2-a1500dd0507f-000000%2FYsTkSdt_0BwtV399Qs_g83HnOCMQzcfnwHRJndhMUv0%3D258&data=05%7C02%7Ce.versace%40qmul.ac.uk%7C9a1139c9f29e4cc918d308de9f56133c%7C569df091b01340e386eebd9cb9e25814%7C0%7C0%7C639123389672009006%7CUnknown%7CTWFpbGZsb3d8eyJFbXB0eU1hcGkiOnRydWUsIlYiOiIwLjAuMDAwMCIsIlAiOiJXaW4zMiIsIkFOIjoiTWFpbCIsIldUIjoyfQ%3D%3D%7C0%7C%7C%7C&sdata=nbgUNc1U%2FGpovXL4Dd6N71ARJlPcwhCv11Hr5Ect%2F%2FA%3D&reserved=0)

Done

2.   We noted in your submission details that a portion of your manuscript may have been presented or published elsewhere. We have a preprint on psyarxiv: <https://osf.io/preprints/psyarxiv/9tn7h_v1>. Please clarify whether this [conference proceeding or publication] was peer-reviewed and formally published. If this work was previously peer-reviewed and published, in the cover letter please provide the reason that this work does not constitute dual publication and should be included in the current manuscript.

Dear Editor, the preprint on psyarxiv is not peer-reviewed or formally published.

3. We note that the grant information you provided in the ‘Funding Information’ and ‘Financial Disclosure’ sections do not match. When you resubmit, please ensure that you provide the correct grant numbers for the awards you received for your study in the ‘Funding Information’ section.

This is the full financial disclosure: “This study was funded by the Economic and Social Research Council research grant ES/V015354/1 granted to EV, MA and DM. EV was also funded by the Royal Society Leverhulme Trust fellowship SRF\R1\21000155. DM acknowledges the support of the National Institute for Health Research Barts Biomedical Research Centre (NIHR203330).”

4. Please expand the acronym “ESRC” (as indicated in your financial disclosure) so that it states the name of your funders in full. This information should be included in your cover letter; we will change the online submission form on your behalf.

We have now reported the full name as Economic and Social Research Council. We have now added the financial disclosure to the cover letter.

5. Please remove your figures from within your manuscript file, leaving only the individual TIFF/EPS image files, uploaded separately. These will be automatically included in the reviewers’ PDF.

Done.

6. We note that Figure 1 includes an image of a [patient / participant / in the study].
As per the PLOS ONE policy (http://journals.plos.org/plosone/s/submission-guidelines#loc-human-subjects-research) on papers that include identifying, or potentially identifying, information, the individual(s) or parent(s)/guardian(s) must be informed of the terms of the PLOS open-access (CC-BY) license and provide specific permission for publication of these details under the terms of this license. Please download the Consent Form for Publication in a PLOS Journal (http://journals.plos.org/plosone/s/file?id=8ce6/plos-consent-form-english.pdf). The signed consent form should not be submitted with the manuscript, but should be securely filed in the individual's case notes. Please amend the methods section and ethics statement of the manuscript to explicitly state that the patient/participant has provided consent for publication: “The individual in this manuscript has given written informed consent (as outlined in PLOS consent form) to publish these case details”. If you are unable to obtain consent from the subject of the photograph, you will need to remove the figure and any other textual identifying information or case descriptions for this individual.

The figure shows the teacher/model, not a patient or participant. The form you suggest using does not seem relevant in our case. We are waiting for the input from PLOS on how to best proceed.

7. [Please include captions for your Supporting Information files at the end of your manuscript, and update any in-text citations to match accordingly. Please see our Supporting Information guidelines for more information: http://journals.plos.org/plosone/s/supporting-information.](https://eur01.safelinks.protection.outlook.com/?url=http%3A%2F%2Ftrack.editorialmanager.com%2FCL0%2Fhttp%3A%252F%252Fjournals.plos.org%252Fplosone%252Fs%252Fsupporting-information.%2F1%2F010f019dae1529c3-baf455ed-87d7-4be6-a0b2-a1500dd0507f-000000%2FrvzebhP-mhVUAsxCS7aZN5KjTWoa03cjY5WoI-9SqgI%3D258&data=05%7C02%7Ce.versace%40qmul.ac.uk%7C9a1139c9f29e4cc918d308de9f56133c%7C569df091b01340e386eebd9cb9e25814%7C0%7C0%7C639123389672017077%7CUnknown%7CTWFpbGZsb3d8eyJFbXB0eU1hcGkiOnRydWUsIlYiOiIwLjAuMDAwMCIsIlAiOiJXaW4zMiIsIkFOIjoiTWFpbCIsIldUIjoyfQ%3D%3D%7C0%7C%7C%7C&sdata=nGtXzyugQT6PKukwco20kG6kvaVPFyY3T7UfXah0SXM%3D&reserved=0)

Done.

8. If the reviewer comments include a recommendation to cite specific previously published works, please review and evaluate these publications to determine whether they are relevant and should be cited. There is no requirement to cite these works unless the editor has indicated otherwise. 
Reviewers do not recommend citing any specific work.

**Reviewers' comments:**

Reviewer's Responses to Questions

**Comments to the Author**
1. Is the manuscript technically sound, and do the data support the conclusions?

The manuscript must describe a technically sound piece of scientific research with data that supports the conclusions. Experiments must have been conducted rigorously, with appropriate controls, replication, and sample sizes. The conclusions must be drawn appropriately based on the data presented.

Reviewer #1: Partly

2. Has the statistical analysis been performed appropriately and rigorously?

Reviewer #1: Yes

3. Have the authors made all data underlying the findings in their manuscript fully available?

The [PLOS Data policy](https://eur01.safelinks.protection.outlook.com/?url=http%3A%2F%2Ftrack.editorialmanager.com%2FCL0%2Fhttp%3A%252F%252Fwww.plosone.org%252Fstatic%252Fpolicies.action%2523sharing%2F1%2F010f019dae1529c3-baf455ed-87d7-4be6-a0b2-a1500dd0507f-000000%2FuqrXym32x7vivsKWOY4Wf2pgvjnyNUsi3I1FmGMras4%3D258&data=05%7C02%7Ce.versace%40qmul.ac.uk%7C9a1139c9f29e4cc918d308de9f56133c%7C569df091b01340e386eebd9cb9e25814%7C0%7C0%7C639123389672027139%7CUnknown%7CTWFpbGZsb3d8eyJFbXB0eU1hcGkiOnRydWUsIlYiOiIwLjAuMDAwMCIsIlAiOiJXaW4zMiIsIkFOIjoiTWFpbCIsIldUIjoyfQ%3D%3D%7C0%7C%7C%7C&sdata=xTOcIiQGMCa1EvPIns%2B9XxZ47cHkU0IMlKm9g4ktWlU%3D&reserved=0) requires authors to make all data underlying the findings described in their manuscript fully available without restriction, with rare exception (please refer to the Data Availability Statement in the manuscript PDF file). The data should be provided as part of the manuscript or its supporting information, or deposited to a public repository. For example, in addition to summary statistics, the data points behind means, medians and variance measures should be available. If there are restrictions on publicly sharing data—e.g. participant privacy or use of data from a third party—those must be specified.

Reviewer #1: Yes

4. Is the manuscript presented in an intelligible fashion and written in standard English?

PLOS ONE does not copyedit accepted manuscripts, so the language in submitted articles must be clear, correct, and unambiguous. Any typographical or grammatical errors should be corrected at revision, so please note any specific errors here.

Reviewer #1: Yes

**5. Review Comments to the Author**

Please use the space provided to explain your answers to the questions above. You may also include additional comments for the author, including concerns about dual publication, research ethics, or publication ethics. (Please upload your review as an attachment if it exceeds 20,000 characters)

Reviewer #1: Summary of the study
This manuscript examines whether the visual perspective of an instructor during video-based dance teaching influences choreography learning and performance. The topic is interesting, the question is practically relevant, and the study has several strengths, including preregistration, ecological relevance, and expert-rated performance measures. The results suggest that the Back view condition was associated with better performance than the two Front-view conditions, with the clearest support for movement accuracy and some indication that less-trained dancers benefited more.

We thank the Reviewer for the appreciation of our work.

At the same time, the manuscript currently overstates some of its conclusions, especially with respect to cognitive load as a demonstrated mechanism and the consistency of the interaction with expertise. My main concerns are therefore interpretive rather than fatal. I think the paper could become substantially stronger with clearer alignment between the empirical evidence and the claims made in the title, abstract, discussion, and conclusion.

We have carefully addressed these concerns, with a clearer interpretation and alignment between empirical evidence and claims in all sections, as detailed below point by point.

Overall Assessment
The manuscript addresses a meaningful question in dance pedagogy and observational motor learning. The experimental manipulation is sensible, and the core finding (i.e., an advantage of Back view instruction) is potentially useful. However, the manuscript is currently stronger as a study of instructional perspective and choreography learning than as a study demonstrating that dance learning was improved by reducing cognitive load. The authors should temper mechanistic claims, clarify the strength of their evidence, and address several analytical and interpretive issues.

As requested by the Reviewer, we have moderated mechanistic claims, better clarified the strength of evidence (including adding more analyses), and addressed all several analytical and interpretation issues as requested, in all sections, as detailed below point by point.

Recommendations for the authors (major comments)

1. The central mechanistic claim about cognitive load is stronger than the evidence currently supports. This is the main issue throughout the manuscript, including the title, abstract, aims and hypotheses, and discussion. The study is framed as testing cognitive load, and the title explicitly states: “Enhancing dance learning by reducing cognitive load …”. However, in the methods and results sections shared, I do not see a direct measure of cognitive load. Instead, the study manipulates instructional perspective and measures dance performance, then interprets the pattern through cognitive load theory. That theoretical framing is reasonable, but it is not equivalent to demonstrating the mechanism. For example, in the Discussion the authors write that the results “largely support the hypothesis of the lower cognitive load for the Back view.” Based on the reported analyses, the results support the claim that Back view improved performance, but they only support a cognitive-load account indirectly. The findings may also be explained in terms of: (a) reduced visuomotor transformation demands; (b) lower left-right mapping difficulty, (c) greater perceptual compatibility between observed and executed movement. These explanations are related to cognitive load, but not identical to directly measuring it.

Recommendation: The authors should revise the title, abstract, and discussion so that the findings are described as consistent with a reduced extraneous-load interpretation, unless direct cognitive-load measurements are reported.

We have revised our manuscript to clarify our theoretical framework. We agree that visuomotor transformation and left-right mapping difficulty describe the specific cognitive processes involved, and that “the findings are described as consistent with a reduced extraneous-load interpretation.” We use this interpretation because these processes (visuomotor transformation demands, left-right mapping difficulty; perceptual compatibility between observed and executed movement) are core components of extraneous cognitive load, as they reflect mental processing imposed by the instructional format (e.g., viewing perspective) rather than the intrinsic difficulty of the task. As Sweller et al. (1998) note: “extraneous cognitive load is unnecessary cognitive load and can be altered by instructional interventions. Extraneous cognitive load is determined by the instructional design … extraneous cognitive load reflects the effort required to process poorly designed instruction …,” and “extraneous cognitive load … provides the core of cognitive load theory and is under the direct control of instructional designers.” In this framework, “mental integration likely imposes a heavy, extraneous cognitive load… The load is extraneous because it is caused entirely by the format of the instruction rather than by the intrinsic characteristics of the material.” Our framework is also consistent with more recent work (e.g. Orru and Longo, 2019), which similarly defines extraneous load as arising from instructional designs that “hamper the construction of schema in working memory,” and emphasises that its reduction is a central goal of Cognitive Load Theory. We have now better specified this theoretical framework in the paper, starting from the title, as suggested:

a) Title: “*Enhancing dance learning by optimising instructional perspective: A preregistered study on the effect of dancers’ viewing perspective, cognitive load and expertise”*

b) Abstract: “*Drawing on cognitive load theory, we investigated how the requests imposed by different instructional perspectives affect learning and performance. We compared the effects of Back view (first-person), Front mirror view (third-person mirrored), and Front opposite view (third-person) between dancers*.”

c) Introduction: “*Second, the* ***extraneous load*** *refers to* ***how the instructional material is presented (rather than to the intrinsic characteristics of the learned material), with the effect of hindering or supporting learning*** *(30). For instance, slower instructions enhance learning in tasks with medium and high complexity (31).* ***In this work, we study the effect of the extraneous load imposed by the instructional point of view. An efficient instructional design should reduce the extraneous cognitive load*** *(11,12). We used a between-subjects design to investigate the impact of three different teacher viewing perspectives on dance learning (see Figure 1): (1) first-person view from behind the teacher (Back view, hypothesised to impose the lowest extraneous cognitive load, Fig. 1a), (2) third-person view mirrored, namely the viewpoint of someone facing the mirror image of the instructor (Front mirror view; expected to impose a moderate extraneous cognitive load, Fig. 1b), and (3) third-person view without a mirror image (Front opposite: expected to impose the highest cognitive load, Fig. 1c). We focused on a choreography of medium complexity, that required a minimum of three years of ballet training to be learnt in two teaching sessions of approximately 60 minutes*.”

d) Discussion: *“We investigated how different visual perspectives of a dance instructor (first-person Back view, third-person Front mirror, and third-person Front opposite) influence dance learning and performance, as moderated by participants' training experience. This addresses the issue of extraneous cognitive load (cognitive load dependent on the instructional layout) in motor learning.”*

The Reviewer also makes a distinction between cognitive load and measures of dance performance. We have now better specified in the Methods: “***Cognitive load was not directly measured; instead, we interpreted differences in performance across conditions as reflecting variation in processing demands induced by the instructional perspective****. The use of primary task performance to infer cognitive load dynamics is validated methodology (31,41,42)*.

2. The results support the Back-view hypothesis more clearly than the full hypothesized gradient across all three conditions. The manuscript predicts a graded pattern: Back = lowest load (best performance); Front mirror = intermediate; Front opposite = highest load (worst performance). However, the reported results do not support the second part of this prediction. The key comparison between Front mirror and Front opposite appears null. This is already noted by the authors, but in the Discussion it is treated mainly as a power issue or a consequence of dancers’ prior exposure to both perspectives. I think this result deserves more direct theoretical treatment. The data seem to support a simpler conclusion: Back view is advantageous and the two front-view conditions are not meaningfully distinguishable in this study. That weakens the claim that the manipulation works through a fine-grained gradient of mental transformation demands. It may instead suggest that the main distinction is between body-aligned Back view and front-facing instruction, rather than between two levels of front-view difficulty.

Recommendation: The authors should address the failure of the Front mirror > Front opposite hypothesis more explicitly and consider whether the data support a simpler theoretical interpretation.

Thank you for the opportunity to better specify the alternative explanations for the lack of significant differences between the front views. We have now fully acknowledged this aspect in the Discussion, starting from the simpler theoretical interpretation mentioned by the reviewer:*“Overall, this pattern is consistent with* *the hypothesis of the lower cognitive load for the Back view, as we observed an enhanced performance either as a significant effect or as a trend across all variables: accuracy of movements, spatial skills, and dynamics/rhythm. Front views posed greater challenges, particularly for dancers with lower training levels. The data did not provide full support for the predicted differentiation between the two front conditions, as performance was not significantly different between Front mirror and Front opposite.* ***This suggests that the primary distinction captured in this study may be between body-aligned (Back) and front-facing perspectives, rather than between graded levels of front-view difficulty****.* *However, the lack of significant differences between the two front views could also be attributed to the reduced sample size, which was impacted by the recruitment challenges following the ease of the COVID-19 restrictions, and that forced us to reduce the recruitment targets. Indeed, a limitation of our study is the reduced sample size, as recruiting dancers specifically trained in ballet for individual sessions across multiple days has been a challenge. Hence,* one possibility is that limited statistical power or participants’ prior exposure to both front-facing perspectives reduced sensitivity to detect differences between these conditions. *Alternatively, or additionally, the lack of significant differences between mirror and opposite front views can reflect the current exposure of dancers to both perspectives in their in-person dance training, or to the widespread exposure to third-perspective videos via social media and digital tools widely available. Future studies should clarify how different types of training affect cognitive load longitudinally.*

3. The interaction with expertise is only partially supported and should not be described too strongly. The manuscript’s second main theoretical claim concerns expertise reversal: less experienced dancers should benefit more from a low-load instructional perspective. However, the evidence is mixed: for Accuracy of movements, the interaction between point of view and training is significant; for Spatial skills, the interaction is not significant; for Dynamics and rhythmical accuracy, the interaction is trend-level only. Despite this, the Discussion states that “the effect of the point of view was strongly affected by the expertise of dancers, as shown by the significant interactions on point of view and training.” This overstates the evidence.
Recommendation: The authors should revise the Discussion to state that the moderation by expertise was clearest for movement accuracy, weaker for the other outcomes, and therefore provides partial rather than uniform support for the expertise-reversal account.

We thank the reviewer for the opportunity to revise the Discussion and refine our claims, contextualising the results for specific dependent variables, and mentioning the partial support: *“The effect of Point of View and Training is better understood when considering their interaction, at least for the movement accuracy. An interaction between Point of View and Training was observed for movement accuracy, whereas this pattern was weaker or not statistically robust for the other outcomes. The modulation by expertise was clearest for movement accuracy. In the contest of movement accuracy, the effect of the point of view was strongly affected by the expertise of dancers, as shown by the significant interactions on Point of View and Training. This suggests that, in front of the same choreography, different strategies might be needed for dancers with different levels of expertise. While the fact that the Back view appeared to particularly facilitate less experienced dancers suggests that the benefit of a first-person perspective is higher in less experienced dancers, only few experienced dancers have been tested on the Back point of view****. Taken together, these findings provide some partial support for an expertise-reversal account****.”; …. “Taken together, our results suggest that first-person perspectives may represent a useful strategy to adapt instructions to learner expertise and facilitate the task.”*

4. Sample size, recruitment changes, and power need more explicit discussion
The final analyzed sample is small (31 participants across three groups), particularly given: the three-condition design, the inclusion of a continuous expertise moderator, the emphasis on interaction effects. The manuscript notes that the original recruitment and power rationale changed because the target population became harder to access after COVID. This is understandable, but it is important because the study moved from a more homogeneous high-expertise sample to a more heterogeneous sample with different backgrounds and current training levels.
Recommendation: The authors should more explicitly acknowledge the limitations of precision and power, especially for the interaction terms and for detecting differences between Front mirror and Front opposite.

We have taken this opportunity to clarify the aspects indicated by the Reviewer:

**Sample size**, in the Methods section: “*After ethical approval from the Queen Mary University Research Ethics Committee (QMREC2020/34), we recruited voluntary participants among active dancers aged 18 years or older (range 18-43 years old, M=23, SD=5.15), with at least 3 years of ballet training****. Dancers were recruited using a convenience sampling approach via advertisements at three London-based vocational dance schools and through affiliated dance teachers.*** *Recruitment started on June 2021 and ended on January 2023. We recruited a total of 32 participants (27 females and 5 males). One participant was excluded due to a procedural error (incorrect presentation of viewpoints), resulting in a final analysed sample of 31 participants (26 females, 5 males). Participants were drawn from three different backgrounds: vocational ballet schools (n=4), vocational contemporary dance schools (n=8), and independent dancers not enrolled in vocational training (n=19). Participants were stratified by sex and randomly assigned to different experimental conditions*.”

and in the Discussion: “*Indeed, a limitation of our study is the reduced sample size, as recruiting dancers specifically trained in ballet for individual sessions across multiple days has been a challenge. Hence, one possibility is that limited statistical power or participants’ prior exposure to both front-facing perspectives reduced sensitivity to detect differences between these conditions*”.

**Recruitment changes and power**: In the Methods: *“The pre-registered design targeted a relatively homogeneous sample of highly trained dancers, with an expected small effect size (f=0.26; α=0.05, power=0.8; target N≤147). As shortly before data collection lockdown restrictions were lifted and in-person teaching resumed, recruitment opportunities were substantially reduced for the vocational students initially targeted. To address this challenge, we broadened recruitment to include dancers with a minimum experience of three years of ballet, which was needed to match the requirements of the choreography. While this introduced greater variability in participants’ expertise, it increased the expected effect size and allowed us to explore a key aspect of cognitive load theory: the differences in learning between different levels of expertise.*”

We also specified in a dedicated section in the Methods “***Sample size and sample composition*** *This study was originally planned and funded as an acute response to the lockdown training experienced during the Covid-19 pandemic, focusing on isolated individual vocational dance students (16-18 years), with a similar level of training. However, as the lockdown was lifted, vocational dancers were extremely busy with in-person group classes, so that only 4 vocational dancers could enrol in this experiment. Hence, we have been forced to reduce the sample size and to open-up registration to non-vocational dancers. As inclusion criteria, we required participants to be currently active in dance and to have at least 3 years of experience in ballet. Because we included dancers with levels and type of experience, we used training practice as a proxy for expertise. We measured training as the number of hours spent in physical training every week. Accounting for expertise is essential, as it can dramatically affect the cognitive load (see Introduction)*.” and in the Discussion “*Overall, this pattern is consistent with* *the hypothesis of the lower cognitive load for the Back view, as we observed an enhanced performance either as a significant effect or as a trend for this condition across all variables: accuracy of movements, spatial skills, and dynamics/rhythm. Front views posed greater challenges, particularly for dancers with lower training levels. We did not observe significant differences between the two front views. This could be attributed to the reduced sample size, which was impacted by the recruitment challenges following the ease of the COVID-19 restrictions, and that forced us to reduce the recruitment targets. Indeed, a limitation of our study is the reduced sample size, as recruiting dancers specifically trained in ballet for individual sessions across multiple days is a challenge. Alternatively, or additionally, the lack of significant differences between mirror and opposite front views can reflect the current exposure of dancers to both perspectives in their in-person dance training, or to the widespread exposure to third-perspective videos via social media and digital tools widely available. Future studies should clarify how different types of training affect cognitive load longitudinally*.”

The Discussion has been **toned down, as requested**: “*The data* ***did not provide full support for the predicted differentiation between the two front conditions, as performance was not significantly different between Front mirror and Front opposite views****. This suggests that the primary distinction captured in this study may be between body-aligned (Back) and front-facing perspectives, rather than between graded levels of front-view difficulty. However, the lack of significant differences between the two front views could also be attributed to the reduced sample size, which was impacted by the recruitment challenges following the ease of the COVID-19 restrictions, and that forced us to reduce the recruitment targets. Indeed, a limitation of our study is the reduced sample size, as recruiting dancers specifically trained in ballet for individual sessions across multiple days has been a challenge. Hence, one possibility is that limited statistical power or participants’ prior exposure to both front-facing perspectives reduced sensitivity to detect differences between these conditions. Alternatively, or additionally, the lack of significant differences between mirror and opposite front views can reflect the current exposure of dancers to both perspectives in their in-person dance training, or to the widespread exposure to third-perspective videos via social media and digital tools widely available. Future studies should clarify how different types of training affect cognitive load longitudinally.” and “Taken together, our results suggest that first-person perspectives may represent a useful strategy to adapt instructions to learner expertise and facilitate the task.”*

5. The dependent variables are highly collinear, which affects interpretation. The manuscript reports very high correlations among Accuracy of movements, Spatial skills, and Dynamics and rhythmical accuracy (approx. r = 0.90). This suggests overlap among the outcomes. Analyzing them separately is not necessarily wrong, especially if they are conceptually distinct. However, such strong collinearity means the reader should not interpret three significant models as three independent replications of the same effect. In addition, I did not see discussion of correction for multiple testing. Recommendation: The authors should either justify more clearly why separate analyses were retained despite very high overlap, report a composite performance score in addition to the separate outcomes, or discuss the implications of this collinearity more explicitly.

We have now **more clearly justified the importance of reporting the collinear variables**, as these variables are often used together in dance assessment but there are few related quantitative studies. Our report will be useful to build quantitative comparison in dance science, a field that suffers from limited availability in quantitative data: “*This strong multicollinearity likely reflects a common underlying construct in performance. For completeness, we report results of all three variables, as they are potentially independent (e.g., accurate movements can be performed in the wrong spatial location, at the wrong time). As these measures are often used together in dance assessments, it will be informative to provide full reporting of all metrics to facilitate future meta-analyses and establish standardized benchmarks in dance science.”*

**Minor recommendations for the authors (minor comments).**
1. Clarify the study design earlier and more explicitly. In the abstract, the phrasing may give the impression that participants learned in all three perspectives, which could be interpreted as a within-subjects design. However, the Methods clarify that participants were randomly assigned to one of three conditions.

Thank you for the opportunity to better specify the experimental design. We have improved both the Abstract: “*We compared the effects of Back view (first-person), Front mirror view (third-person mirrored), and Front opposite view (third-person) between dancers.*”

and the Methods: *“We used a between-subjects design to investigate the impact of three different teacher viewing perspectives on dance learning (see Figure 1): …*”.

2. The initial ICC analysis based on only three participants rated by all five experts is informative as a preliminary check, but it should not be emphasized too strongly.

As suggested, we have removed the section “To ensure a robust starting point” related to the initial ICC analysis and better specified: “*We initially assessed the inter-rater reliability using Intraclass Correlation Coefficients (ICC) (42,43), as visualised in Figure 2. We first examined a subset of three participants (P12, P14, P15) who had been rated by all five experts.*”

3. The title should be reconsidered if cognitive load was not directly measured. As written, it implies a demonstrated mechanism.

Following the Reviewer suggestion, we have changed the title to acknowledge the direct role of the instructional perspective, our preregistered hypothesis based on cognitive load, and the role of viewing perceptive and expertise: “*Enhancing dance learning by optimising instructional perspective: A preregistered study on the effect of dancers’ viewing perspective, cognitive load and expertise*”.

4. In the abstract and discussion, causal statements such as “enhance learning by reducing cognitive load” should be softened.

We have followed these suggestions. **Abstract:** “*These findings suggest that first-person perspectives may support motor learning, with performance patterns consistent with reduced cognitive load”*; **Discussion:** “*The data did not provide full support for the predicted differentiation between the two front conditions, as performance was not significantly different between Front mirror and Front opposite views. This suggests that the primary distinction captured in this study may be between body-aligned (Back) and front-facing perspectives, rather than between graded levels of front-view difficulty. However, the lack of significant differences between the two front views could also be attributed to the reduced sample size, which was impacted by the recruitment challenges following the ease of the COVID-19 restrictions, and that forced us to reduce the recruitment targets. Indeed, a limitation of our study is the reduced sample size, as recruiting dancers specifically trained in ballet for individual sessions across multiple days has been a challenge. Hence, one possibility is that limited statistical power or participants’ prior exposure to both front-facing perspectives reduced sensitivity to detect differences between these conditions. Alternatively, or additionally, the lack of significant differences between mirror and opposite front views can reflect the current exposure of dancers to both perspectives in their in-person dance training, or to the widespread exposure to third-perspective videos via social media and digital tools widely available. Future studies should clarify how different types of training affect cognitive load longitudinally*.”; “*Our findings suggest that the lack of direct experience with the face and gestures of the instructor as seen mostly from the back, does not substantially hinder learning under these conditions*.” ; “*The effect of Point of View and Training is better understood when considering their interaction, at least for the movement accuracy. An interaction between Point of View and Training was observed for movement accuracy, whereas this pattern was weaker or not statistically robust for the other outcomes. The modulation by expertise was clearest for movement accuracy. In the contest of movement accuracy, the effect of the point of view was strongly affected by the expertise of dancers, as shown by the significant interactions on Point of View and Training. This suggests that, in front of the same choreography, different strategies might be needed for dancers with different levels of expertise. While the fact that the Back view appeared to particularly facilitate less experienced dancers suggests that the benefit of a first-person perspective is higher in less experienced dancers, only few experienced dancers have been tested on the Back point of view. Taken together, these findings provide some partial support for an expertise-reversal account.”*

5. The participant-background description in Section 2.2 is somewhat hard to parse and would benefit from clearer presentation.

Thank you for giving us the opportunity to clarify and streamline this section: “*After ethical approval from the Queen Mary University Research Ethics Committee (QMREC2020/34), we recruited voluntary participants among active dancers aged 18 years or older (range 18-43 years old, M=23, SD=5.15), with at least 3 years of ballet training****. Dancers were recruited using a convenience sampling approach via advertisements at three London-based vocational dance schools and through affiliated dance teachers.*** *Recruitment started on June 2021 and ended on January 2023. We recruited a total of 32 participants (27 females and 5 males). One participant was excluded due to a procedural error (incorrect presentation of viewpoints), resulting in a final analysed sample of 31 participants (26 females, 5 males). Participants were drawn from three different backgrounds: vocational ballet schools (n=4), vocational contemporary dance schools (n=8), and independent dancers not enrolled in vocational training (n=19). Participants were stratified by sex and randomly assigned to different experimental conditions. The pre-registered design targeted a relatively homogeneous sample of highly trained dancers, with an expected small effect size (f=0.26; α=0.05, power=0.8; target N≤147). As shortly before data collection lockdown restrictions were lifted and in-person teaching resumed, recruitment opportunities were substantially reduced for the vocational students initially targeted. To address this challenge, we broadened recruitment to include dancers with a minimum experience of three years of ballet, which was needed to match the requirements of the choreography. While this introduced greater variability in participants’ expertise, it increased the expected effect size and allowed us to explore a key aspect of cognitive load theory: the differences in learning between different levels of expertise.”*

6. The manuscript should state the final analyzed sample once and consistently.

We have now clearly specified this once: “We recruited a total of 32 participants (27 females and 5 males). One participant was excluded due to a procedural error (incorrect presentation of viewpoints), resulting in a final analysed sample of 31 participants (26 females, 5 males).”

7. In the Results, the distinction between model-level significance, coefficient significance, and pairwise comparisons should be made as clearly as possible.

Thank you for giving us the opportunity to better clarify these aspects. We now keep explicitly separated the different significance levels, coefficient level and pairwise comparisons.

“***Accuracy of movements*** *To examine the effect of Point of View and Training on accuracy of movements, we fitted a linear model including main effects and their interaction (Figure 3a). The overall model was significant, F_5,25_=4.33, p=0.006, explaining 36% of the variance (adjusted R²=0.36). At the effect level, we observed a significant main effect of Training (F_1,25_=11.463, p=0.002), Point of View (F_2,25_=6.093, p=0.007) and their interaction (F_2,25_=3.459, p=0.047). At the* ***coefficient level*** *(Table 1), relative to the Front opposite condition, the Back condition showed higher accuracy (β=2.48, p=0.003), whereas the Front mirror condition showed lower accuracy (β=-1.92, p=0.026). Training level was positively associated with accuracy (β=0.085, p=0.002). These* ***coefficients a****re consistent with the observed interaction.*

*To further characterise the interaction between Training and Point of View, we examined* ***simple slopes*** *of Training for each condition. Training was not reliably associated with accuracy in the Back condition (β=-0.003, SE=0.044, 95% CI [-0.094, 0.088]), whereas it was positively associated with accuracy in both the Front mirror condition (β=0.161, SE=0.045, 95% CI [0.069, 0.252]) and the Front opposite condition (β=0.097, SE=0.042, 95% CI [0.011, 0.183]).* ***Pairwise comparisons*** *of these slopes indicated that the effect of Training was significantly stronger in the Front mirror condition than in the Back condition (Δβ=0.164, SE=0.063, p=0.039, Tukey-adjusted). Differences between the Back and Front opposite conditions (p=0.246) and between the two Front conditions (p=0.558) were not statistically significant. The full table of coefficients is shown in Table 1.* “

“***Spatial skills*** *To examine the effect of Point of View and Training on spatial skills, we fitted a linear model including main effects and their interaction (Figure 3b). The* ***overall model*** *was significant, F₅,₂₅=2.95, p=0.032, explaining 24% of the variance (adjusted R²=0.24).* ***At the effect level****, we observed a significant main effect of Training (F₁,₂₅=7.82, p=0.010) and Point of View (F₂,₂₅=3.97, p=0.032), but their interaction was not significant (F₂,₂₅=1.99, p=0.158).*

***At the coefficient level*** *(Table 2), relative to the Front opposite condition, the Back condition showed higher spatial skills (β=2.04, p=0.010), whereas the Front mirror condition did not differ significantly (β=-1.41, p=0.092). Training level was positively associated with spatial skills (β=0.070, p=0.010). The interaction terms were not significant (although showed the same trends observed for accuracy of movement), indicating that the effect of Training did not differ reliably across perspectives. The full table of coefficients is shown in Table 2.”*

“***Dynamics and rhythmical accuracy*** *To examine the effect of Point of View and Training on dynamics and rhythmic accuracy, we fitted a linear model including main effects and their interaction (Figure 3c). The* ***overall model*** *was significant, F₅,₂₅=5.05, p=0.003, explaining 38% of the variance (adjusted R²=0.38).* ***At the effect level****, we observed a significant main effect of Training (F₁,₂₅=10.17, p=0.004) and Point of View (F₂,₂₅=4.99, p=0.015). There was also a trend toward an Interaction between Training and Point of View (F₂,₂₅=2.65, p=0.091).* ***At the coefficient level*** *(Table 3), relative to the Front opposite condition, the Back condition showed higher dynamics and rhythmical accuracy (β=2.54, p=0.005), whereas the Front mirror condition did not differ significantly (β=-1.79, p=0.057). Training level was positively associated with performance (β=0.088, p=0.004). The full table of coefficients is shown in Table 3.”*

8. Where effect sizes are discussed despite confidence intervals including zero, the wording should remain cautious.

Thank you for this suggestion, we have now made more explicit the need for remaining cautious in the interpretation in the Results “Although the pairwise comparisons were not significant, effect size estimates suggested a moderate advantage for the Back view over both Front perspectives: Cohen’s d≈0.75–0.79 for the Back vs Front conditions, whereas Cohen’s d≈0.040 for the Front mirror vs Front opposite difference. **Given that these estimates were associated with non-significant comparisons, they should be interpreted cautiously, as do not provide conclusive evidence for differences between conditions**.” and in several parts of the Discussion: “*Overall, this pattern is consistent with* *the hypothesis of the lower cognitive load for the Back view, as we observed an enhanced performance either as a significant effect or as a trend for this condition across all variables: accuracy of movements, spatial skills, and dynamics/rhythm*.”; “*Our findings suggest that the lack of direct experience with the face and gestures of the instructor as seen mostly from the back, does not substantially hinder learning under these conditions*.”, “*While the fact that the Back view appeared to particularly facilitate less experienced dancers suggest*s”; “*Taken together, our results suggest that first-person perspectives may represent a useful strategy to adapt instructions to learner expertise and facilitate the task.*”.

9. The Discussion would benefit from more explicit separation between: findings directly supported by the data; theory-based interpretation; broader practical speculation.

We have better signposted and separated the different aspects in the Discussion: e.g*. “The data did not provide full support…” signalling findings; “This suggests that…” signaling interpretation; “Overall, our findings indicate that a first-person perspective may support motor sequence learning, when high cognitive load is involved. By confirm high inter-rater reliability in performance, we validated a digital tool for the analysis and assessment of motor sequences. These outcomes may have theoretical and practical implications for the advancement of instructional design, the practice and teaching of dance.”*

10. The practical implications are interesting, but the manuscript should avoid overgeneralizing from adult dancers with ballet training to all sports, children, or all motor-sequence learning contexts.

We have rephrased the Discussion to avoid the risk of overgeneralisatuon “*These outcomes may have theoretical and practical implications for the advancement of instructional design, the practice and teaching of dance. Future studies should assess the generalisability to other disciplines in performing arts and sports that require complex sequence learning, both in video/online and in-person settings”.*

6. PLOS authors have the option to publish the peer review history of their article ([what does this mean?](https://eur01.safelinks.protection.outlook.com/?url=https%3A%2F%2Ftrack.editorialmanager.com%2FCL0%2Fhttps%3A%252F%252Fjournals.plos.org%252Fplosone%252Fs%252Feditorial-and-peer-review-process%2523loc-peer-review-history%2F1%2F010f019dae1529c3-baf455ed-87d7-4be6-a0b2-a1500dd0507f-000000%2FjnPKsYQRESC5s0m8JqMOyJvBSFm-YVAeNHzLW3zKqDs%3D258&data=05%7C02%7Ce.versace%40qmul.ac.uk%7C9a1139c9f29e4cc918d308de9f56133c%7C569df091b01340e386eebd9cb9e25814%7C0%7C0%7C639123389672036985%7CUnknown%7CTWFpbGZsb3d8eyJFbXB0eU1hcGkiOnRydWUsIlYiOiIwLjAuMDAwMCIsIlAiOiJXaW4zMiIsIkFOIjoiTWFpbCIsIldUIjoyfQ%3D%3D%7C0%7C%7C%7C&sdata=TOXAmNYve7Dy5WFv8kVlr2ROYzuvxPLt0zA1%2BRw0eaI%3D&reserved=0)). If published, this will include your full peer review and any attached files.
If you choose “no”, your identity will remain anonymous but your review may still be made public.
**Do you want your identity to be public for this peer review?** For information about this choice, including consent withdrawal, please see our [Privacy Policy](https://eur01.safelinks.protection.outlook.com/?url=https%3A%2F%2Ftrack.editorialmanager.com%2FCL0%2Fhttps%3A%252F%252Fwww.plos.org%252Fprivacy-policy%2F1%2F010f019dae1529c3-baf455ed-87d7-4be6-a0b2-a1500dd0507f-000000%2FwMXwyTQPDNFV0prv6pvAomsyXdBIshbubKolyxrTb7Y%3D258&data=05%7C02%7Ce.versace%40qmul.ac.uk%7C9a1139c9f29e4cc918d308de9f56133c%7C569df091b01340e386eebd9cb9e25814%7C0%7C0%7C639123389672045920%7CUnknown%7CTWFpbGZsb3d8eyJFbXB0eU1hcGkiOnRydWUsIlYiOiIwLjAuMDAwMCIsIlAiOiJXaW4zMiIsIkFOIjoiTWFpbCIsIldUIjoyfQ%3D%3D%7C0%7C%7C%7C&sdata=0HiMvvzmWZMmZhKKrcGs%2BLkJXfTroVHrLNtPKbA0DoE%3D&reserved=0).

Reviewer #1: **Yes:** Olgerta Asko

To ensure your figures meet our technical requirements, please review our figure guidelines: <https://journals.plos.org/plosone/s/figures>

You may also use PLOS’s free figure tool, NAAS, to help you prepare publication quality figures: <https://journals.plos.org/plosone/s/figures#loc-tools-for-figure-preparation>.

NAAS will assess whether your figures meet our technical requirements by comparing each figure against our figure specifications.

Done.
