# Supplementary material for: Enhancing dance learning by optimising instructional perspective: A preregistered study on the effect of dancers’ viewing perspective, cognitive load and expertise
Source: PLoS One. 2026 Sep 16;21(9):e0355860. doi: 10.1371/journal.pone.0355860 (PMC13581001; doi:10.1371/journal.pone.0355860)
Supplement: Peer review history

# Peer review: Decision Letter 0

**DOI:** 10.1371/journal.pone.0355860.r001

**Authors:** Emiliano Cè

20 Apr 2026

Dear Dr. Versace,

Thank you for submitting your manuscript to PLOS ONE. After careful consideration, we feel that it has merit but does not fully meet PLOS ONE’s publication criteria as it currently stands. Therefore, we invite you to submit a revised version of the manuscript that addresses the points raised during the review process.

==============================

**ACADEMIC EDITOR**

Dear Authors,

Your manuscript has been reviewed by an expert in the field. During the review process, several critical issues emerged that should be carefully addressed in the revision phase.

==============================

Please submit your revised manuscript by Jun 04 2026 11:59PM. If you will need more time than this to complete your revisions, please reply to this message or contact the journal office at plosone@plos.org. When you're ready to submit your revision, log on to https://www.editorialmanager.com/pone/ and select the 'Submissions Needing Revision' folder to locate your manuscript file.

- A letter that responds to each point raised by the academic editor and reviewer(s). You should upload this letter as a separate file labeled 'Response to Reviewers'.
- A marked-up copy of your manuscript that highlights changes made to the original version. You should upload this as a separate file labeled 'Revised Manuscript with Track Changes'.
- An unmarked version of your revised paper without tracked changes. You should upload this as a separate file labeled 'Manuscript'.

If you would like to make changes to your financial disclosure, please include your updated statement in your cover letter. Guidelines for resubmitting your figure files are available below the reviewer comments at the end of this letter.

If applicable, we recommend that you deposit your laboratory protocols in protocols.io to enhance the reproducibility of your results. Protocols.io assigns your protocol its own identifier (DOI) so that it can be cited independently in the future. For instructions see: https://journals.plos.org/plosone/s/submission-guidelines#loc-laboratory-protocols. Additionally, PLOS ONE offers an option for publishing peer-reviewed Lab Protocol articles, which describe protocols hosted on protocols.io. Read more information on sharing protocols at https://plos.org/protocols?utm_medium=editorial-email&utm_source=authorletters&utm_campaign=protocols.

As the corresponding author, your ORCID iD is verified in the submission system and will appear in the published article. PLOS supports the use of ORCID, and we encourage all coauthors to register for an ORCID iD and use it as well. Please encourage your coauthors to verify their ORCID iD within the submission system before final acceptance, as unverified ORCID iDs will not appear in the published article. *Only*  the individual author can complete the verification step; PLOS staff *cannot*  verify ORCID iDs on behalf of authors.

We look forward to receiving your revised manuscript.

Kind regards,

Emiliano Cè, Ph.D.

Academic Editor

PLOS One

Journal Requirements:

When submitting your revision, we need you to address these additional requirements.

1. Please ensure that your manuscript meets PLOS ONE's style requirements, including those for file naming. The PLOS ONE style templates can be found at

https://journals.plos.org/plosone/s/file?id=wjVg/PLOSOne_formatting_sample_main_body.pdf and https://journals.plos.org/plosone/s/file?id=ba62/PLOSOne_formatting_sample_title_authors_affiliations.pdf

2.   We noted in your submission details that a portion of your manuscript may have been presented or published elsewhere. We have a preprint on psyarxiv: https://osf.io/preprints/psyarxiv/9tn7h_v1. Please clarify whether this [conference proceeding or publication] was peer-reviewed and formally published. If this work was previously peer-reviewed and published, in the cover letter please provide the reason that this work does not constitute dual publication and should be included in the current manuscript.

3. We note that the grant information you provided in the ‘Funding Information’ and ‘Financial Disclosure’ sections do not match.

When you resubmit, please ensure that you provide the correct grant numbers for the awards you received for your study in the ‘Funding Information’ section.

4. Please expand the acronym “ESRC” (as indicated in your financial disclosure) so that it states the name of your funders in full.

This information should be included in your cover letter; we will change the online submission form on your behalf.

5. Please remove your figures from within your manuscript file, leaving only the individual TIFF/EPS image files, uploaded separately. These will be automatically included in the reviewers’ PDF**.**

6. We note that Figure 1 includes an image of a [patient / participant / in the study].

As per the PLOS ONE policy (http://journals.plos.org/plosone/s/submission-guidelines#loc-human-subjects-research) on papers that include identifying, or potentially identifying, information, the individual(s) or parent(s)/guardian(s) must be informed of the terms of the PLOS open-access (CC-BY) license and provide specific permission for publication of these details under the terms of this license. Please download the Consent Form for Publication in a PLOS Journal (http://journals.plos.org/plosone/s/file?id=8ce6/plos-consent-form-english.pdf). The signed consent form should not be submitted with the manuscript, but should be securely filed in the individual's case notes. Please amend the methods section and ethics statement of the manuscript to explicitly state that the patient/participant has provided consent for publication: “The individual in this manuscript has given written informed consent (as outlined in PLOS consent form) to publish these case details”.

If you are unable to obtain consent from the subject of the photograph, you will need to remove the figure and any other textual identifying information or case descriptions for this individual.

7. Please include captions for your Supporting Information files at the end of your manuscript, and update any in-text citations to match accordingly. Please see our Supporting Information guidelines for more information: http://journals.plos.org/plosone/s/supporting-information.

8. If the reviewer comments include a recommendation to cite specific previously published works, please review and evaluate these publications to determine whether they are relevant and should be cited. There is no requirement to cite these works unless the editor has indicated otherwise.

[Note: HTML markup is below. Please do not edit.]

Reviewers' comments:

Reviewer's Responses to Questions

**Comments to the Author**

1. Is the manuscript technically sound, and do the data support the conclusions?

Reviewer #1: Partly

**********

2. Has the statistical analysis been performed appropriately and rigorously?

Reviewer #1: Yes

**********

3. Have the authors made all data underlying the findings in their manuscript fully available?

The PLOS Data policy

Reviewer #1: Yes

**********

4. Is the manuscript presented in an intelligible fashion and written in standard English?

Reviewer #1: Yes

**********

Reviewer #1: Summary of the study

This manuscript examines whether the visual perspective of an instructor during video-based dance teaching influences choreography learning and performance. The topic is interesting, the question is practically relevant, and the study has several strengths, including preregistration, ecological relevance, and expert-rated performance measures. The results suggest that the Back view condition was associated with better performance than the two Front-view conditions, with the clearest support for movement accuracy and some indication that less-trained dancers benefited more.

At the same time, the manuscript currently overstates some of its conclusions, especially with respect to cognitive load as a demonstrated mechanism and the consistency of the interaction with expertise. My main concerns are therefore interpretive rather than fatal. I think the paper could become substantially stronger with clearer alignment between the empirical evidence and the claims made in the title, abstract, discussion, and conclusion.

Overall Assessment

The manuscript addresses a meaningful question in dance pedagogy and observational motor learning. The experimental manipulation is sensible, and the core finding (i.e., an advantage of Back view instruction) is potentially useful. However, the manuscript is currently stronger as a study of instructional perspective and choreography learning than as a study demonstrating that dance learning was improved by reducing cognitive load. The authors should temper mechanistic claims, clarify the strength of their evidence, and address several analytical and interpretive issues.

Recommendations for the authors (major comments)

1. The central mechanistic claim about cognitive load is stronger than the evidence currently supports. This is the main issue throughout the manuscript, including the title, abstract, aims and hypotheses, and discussion. The study is framed as testing cognitive load, and the title explicitly states: “Enhancing dance learning by reducing cognitive load …”. However, in the methods and results sections shared, I do not see a direct measure of cognitive load. Instead, the study manipulates instructional perspective and measures dance performance, then interprets the pattern through cognitive load theory. That theoretical framing is reasonable, but it is not equivalent to demonstrating the mechanism. For example, in the Discussion the authors write that the results “largely support the hypothesis of the lower cognitive load for the Back view.” Based on the reported analyses, the results support the claim that Back view improved performance, but they only support a cognitive-load account indirectly. The findings may also be explained in terms of: (a) reduced visuomotor transformation demands; (b) lower left-right mapping difficulty, (c) greater perceptual compatibility between observed and executed movement. These explanations are related to cognitive load, but not identical to directly measuring it.

Recommendation: The authors should revise the title, abstract, and discussion so that the findings are described as consistent with a reduced extraneous-load interpretation, unless direct cognitive-load measurements are reported.

2. The results support the Back-view hypothesis more clearly than the full hypothesized gradient across all three conditions

The manuscript predicts a graded pattern: Back = lowest load (best performance); Front mirror = intermediate; Front opposite = highest load (worst performance). However, the reported results do not support the second part of this prediction. The key comparison between Front mirror and Front opposite appears null. This is already noted by the authors, but in the Discussion it is treated mainly as a power issue or a consequence of dancers’ prior exposure to both perspectives. I think this result deserves more direct theoretical treatment. The data seem to support a simpler conclusion: Back view is advantageous and the two front-view conditions are not meaningfully distinguishable in this study. That weakens the claim that the manipulation works through a fine-grained gradient of mental transformation demands. It may instead suggest that the main distinction is between body-aligned Back view and front-facing instruction, rather than between two levels of front-view difficulty.

Recommendation: The authors should address the failure of the Front mirror > Front opposite hypothesis more explicitly and consider whether the data support a simpler theoretical interpretation.

3. The interaction with expertise is only partially supported and should not be described too strongly.

The manuscript’s second main theoretical claim concerns expertise reversal: less experienced dancers should benefit more from a low-load instructional perspective. However, the evidence is mixed: for Accuracy of movements, the interaction between point of view and training is significant; for Spatial skills, the interaction is not significant; for Dynamics and rhythmical accuracy, the interaction is trend-level only. Despite this, the Discussion states that “the effect of the point of view was strongly affected by the expertise of dancers, as shown by the significant interactions on point of view and training.” This overstates the evidence.

Recommendation: The authors should revise the Discussion to state that the moderation by expertise was clearest for movement accuracy, weaker for the other outcomes, and therefore provides partial rather than uniform support for the expertise-reversal account.

4. Sample size, recruitment changes, and power need more explicit discussion

The final analyzed sample is small (31 participants across three groups), particularly given: the three-condition design, the inclusion of a continuous expertise moderator, the emphasis on interaction effects. The manuscript notes that the original recruitment and power rationale changed because the target population became harder to access after COVID. This is understandable, but it is important because the study moved from a more homogeneous high-expertise sample to a more heterogeneous sample with different backgrounds and current training levels.

Recommendation: The authors should more explicitly acknowledge the limitations of precision and power, especially for the interaction terms and for detecting differences between Front mirror and Front opposite.

5. The dependent variables are highly collinear, which affects interpretation.

The manuscript reports very high correlations among Accuracy of movements, Spatial skills, and Dynamics and rhythmical accuracy (approx. r = 0.90). This suggests overlap among the outcomes. Analyzing them separately is not necessarily wrong, especially if they are conceptually distinct. However, such strong collinearity means the reader should not interpret three significant models as three independent replications of the same effect. In addition, I did not see discussion of correction for multiple testing.

Recommendation: The authors should either justify more clearly why separate analyses were retained despite very high overlap, report a composite performance score in addition to the separate outcomes, or discuss the implications of this collinearity more explicitly.

Minor recommendations for the authors (minor comments).

1. Clarify the study design earlier and more explicitly. In the abstract, the phrasing may give the impression that participants learned in all three perspectives, which could be interpreted as a within-subjects design. However, the Methods clarify that participants were randomly assigned to one of three conditions.

2. The initial ICC analysis based on only three participants rated by all five experts is informative as a preliminary check, but it should not be emphasized too strongly.

3. The title should be reconsidered if cognitive load was not directly measured. As written, it implies a demonstrated mechanism.

4. In the abstract and discussion, causal statements such as “enhance learning by reducing cognitive load” should be softened.

5. The participant-background description in Section 2.2 is somewhat hard to parse and would benefit from clearer presentation.

6. The manuscript should state the final analyzed sample once and consistently.

7. In the Results, the distinction between model-level significance, coefficient significance, and pairwise comparisons should be made as clearly as possible.

8. Where effect sizes are discussed despite confidence intervals including zero, the wording should remain cautious.

9. The Discussion would benefit from more explicit separation between: findings directly supported by the data; theory-based interpretation; broader practical speculation.

10. The practical implications are interesting, but the manuscript should avoid overgeneralizing from adult dancers with ballet training to all sports, children, or all motor-sequence learning contexts.

**********

what does this mean?). If published, this will include your full peer review and any attached files.

If you choose “no”, your identity will remain anonymous but your review may still be made public.

**Do you want your identity to be public for this peer review?**  For information about this choice, including consent withdrawal, please see our Privacy Policy

Reviewer #1: **Yes:** Olgerta Asko

**********

[NOTE: If reviewer comments were submitted as an attachment file, they will be attached to this email and accessible via the submission site. Please log into your account, locate the manuscript record, and check for the action link "View Attachments". If this link does not appear, there are no attachment files.]

To ensure your figures meet our technical requirements, please review our figure guidelines: https://journals.plos.org/plosone/s/figures

You may also use PLOS’s free figure tool, NAAS, to help you prepare publication quality figures: https://journals.plos.org/plosone/s/figures#loc-tools-for-figure-preparation.

NAAS will assess whether your figures meet our technical requirements by comparing each figure against our figure specifications.

# Author comment: Author response to Decision Letter 1

**DOI:** 10.1371/journal.pone.0355860.r002

30 Jun 2026

See attached point by point response to reviewers.

Attachment Submitted filename: Response Reviewers PLOS_submission.docx

# Peer review: Decision Letter 1

**DOI:** 10.1371/journal.pone.0355860.r003

**Authors:** Emiliano Cè

28 Jul 2026

Enhancing dance learning by optimising instructional perspective: A preregistered study on the effect of dancers’ viewing perspective, cognitive load and expertise

PONE-D-26-00870R1

Dear Dr. Versace,

We’re pleased to inform you that your manuscript has been judged scientifically suitable for publication and will be formally accepted for publication once it meets all outstanding technical requirements.

Within one week, you’ll receive an e-mail detailing the required amendments. When these have been addressed, you’ll receive a formal acceptance letter and your manuscript will be scheduled for publication.

An invoice will be generated when your article is formally accepted. Please note, if your institution has a publishing partnership with PLOS and your article meets the relevant criteria, all or part of your publication costs will be covered. Please make sure your user information is up-to-date by logging into Editorial Manager at Editorial Manager® and clicking the ‘Update My Information' link at the top of the page. For questions related to billing, please contact billing support.

If your institution or institutions have a press office, please notify them about your upcoming paper to help maximize its impact. If they’ll be preparing press materials, please inform our press team as soon as possible -- no later than 48 hours after receiving the formal acceptance. Your manuscript will remain under strict press embargo until 2 pm Eastern Time on the date of publication. For more information, please contact onepress@plos.org.

Kind regards,

Emiliano Cè, Ph.D.

Academic Editor

PLOS One

Additional Editor Comments (optional):

Reviewers' comments:

Reviewer's Responses to Questions

**Comments to the Author**

Reviewer #1: All comments have been addressed

**********

2. Is the manuscript technically sound, and do the data support the conclusions?

Reviewer #1: Yes

**********

3. Has the statistical analysis been performed appropriately and rigorously?

Reviewer #1: Yes

**********

4. Have the authors made all data underlying the findings in their manuscript fully available?

The PLOS Data policy

Reviewer #1: Yes

**********

5. Is the manuscript presented in an intelligible fashion and written in standard English?

Reviewer #1: Yes

**********

Reviewer #1: The authors have sufficiently addressed the comments and concerns I raised during the first round of review. I am satisfied with the revised manuscript and believe that it is now suitable for publication.

**********

what does this mean?). If published, this will include your full peer review and any attached files.

If you choose “no”, your identity will remain anonymous but your review may still be made public.

**Do you want your identity to be public for this peer review?**  For information about this choice, including consent withdrawal, please see our Privacy Policy

Reviewer #1: **Yes:** Olgerta Asko

**********

# Editor report: Acceptance letter

**DOI:** 10.1371/journal.pone.0355860.r004

**Authors:** Emiliano Cè

PONE-D-26-00870R1

PLOS One

Dear Dr. Versace,

I'm pleased to inform you that your manuscript has been deemed suitable for publication in PLOS One. Congratulations! Your manuscript is now being handed over to our production team.

At this stage, our production department will prepare your paper for publication. This includes ensuring the following:

* All references, tables, and figures are properly cited

* All relevant supporting information is included in the manuscript submission,

* There are no issues that prevent the paper from being properly typeset

You will receive further instructions from the production team, including instructions on how to review your proof when it is ready. Please keep in mind that we are working through a large volume of accepted articles, so please give us a few days to review your paper and let you know the next and final steps.

Lastly, if your institution or institutions have a press office, please let them know about your upcoming paper now to help maximize its impact. If they'll be preparing press materials, please inform our press team within the next 48 hours. Your manuscript will remain under strict press embargo until 2 pm Eastern Time on the date of publication. For more information, please contact onepress@plos.org.

You will receive an invoice from PLOS for your publication fee after your manuscript has reached the completed accept phase. If you receive an email requesting payment before acceptance or for any other service, this may be a phishing scheme. Learn how to identify phishing emails and protect your accounts at https://explore.plos.org/phishing.

If we can help with anything else, please email us at customercare@plos.org.

Thank you for submitting your work to PLOS One and supporting open access.

Kind regards,

PLOS One Editorial Office Staff

on behalf of

Prof. Emiliano Cè

Academic Editor

PLOS One
